# Global, regional, and national burden of non-rheumatic valvular heart disease and Its projections to 2035: comprehensive analysis of the global burden of disease study 2019

**DOI:** 10.3389/fcvm.2025.1445024

**Published:** 2025-08-01

**Authors:** Tao Ying, Qiong Nie, Wei Yan, Han Wang, Jing Wu

**Affiliations:** ^1^ Department of Geriatrics, The Third People’s Hospital of Chengdu, The Affiliated Hospital of Southwest Jiaotong University, College of Medicine, Southwest Jiaotong University, Chengdu, Sichuan, China; ^2^ Department of Cardiology, The Third People’s Hospital of Chengdu, Chengdu, Sichuan, China

**Keywords:** global burden of disease, GBD 2019, non-rheumatic valvular heart disease, epidemiology, projections

## Abstract

**Objective:**

Valvular heart disease has attracted global attention as the next heart epidemic. However, the control effect of non-rheumatic valvular heart disease (NRVHD) is not ideal. We systematically and comprehensively assessed the epidemiological information and attributable risk factors of NRVHD from 1990 to 2019, and projections by 2035.

**Methods:**

Data on NRVHD were from the Global Burden of Disease (GBD) 2019. We analyzed the disease burden by age, sex, and socio-demographic index (SDI) regions from 1990 to 2019 using the Joinpoint regression model. We calculated attributable mortality and disability-adjusted life years (DALYs) associated with potential risk factors using a comparative risk assessment. Additionally, Nordpred age-period-cohort analysis predicted the NRVHD burden for the next 15 years.

**Results:**

From 1990 to 2019, incident, prevalent, and death cases, and DALYs of NRVHD gradually increased globally. The age-standardized incidence (ASIR) and prevalence rate (ASPR) of NRVHD continued to increase, while age-standardized mortality (ASMR) and DALYs rate (ASDR) gradually decreased. Subgroup analysis stratified by age, sex, and SDI regions suggested: (1) The global burden of NRVHD in women is generally decreasing. (2) In people older than 55 years, ASPR and ASIR continued to increase with aging, but the decline of ASMR and ASDR was relatively flat. (3) Despite an overall decline in ASMR and ASDR, the disease burden of NRVHD was highest in high SDI regions. High systolic blood pressure was the most prominent risk factor for NRVHD, with much higher mortality and DALYs than the high-sodium diet and lead exposure. The Nordpred age-period-cohort analysis demonstrated that ASIR (20.28 per 100,000 population) is on an upward trend, and ASMR (2.06 per 100,000 population) and ASDR (33.74 per 100,000 population) are on a downward trend in the next 15 years.

**Conclusion:**

The global burden of NRVHD remains high, especially among older adults and men in high SDI areas. However, ASMR and ASDR have declined for nearly 10 years and will likely continue to do so for the next 15 years. This suggests that current medical interventions and hypertension control strategies have been effective in reducing the NRVHD burden.

## 1 Introduction

Valvular heart disease (VHD) is a major global public health problem and is one of the causes of the rapid increase in cardiovascular morbidity and mortality worldwide (1). VHD mainly includes rheumatic heart disease (RHD) and non-rheumatic valvular heart disease (NRVHD). RHD affects approximately 41 million people, whose prevalence continues to rise in developing countries and among the poor in developed countries (2, 3). Decades ago, most VHD was caused by RHD. However, over the past 40 years, rapid economic development and an aging population have led to a significant decrease in the incidence of RHD, while the incidence of NRVHD has been steadily increasing, particularly among the elderly (4). Patients with NRVHD continue to increase, which imposes an economic and social burden on the world, particularly in high socio-demographic index (SDI) regions as well as developing countries. NRVHD mainly includes aortic, mitral, and tricuspid valve disease. In high SDI regions, for example, aortic and mitral valve disease accounted for nearly 80% of all VHD deaths and the most dominant form of aortic valve disease is calcific aortic stenosis in these countries, characterized by thickening and calcification of the aortic valve (1). In 2019, about 9.4 million patients worldwide had calcific aortic valve disease (CAVD), which is closely related to age, with studies reporting that the prevalence of aortic and mitral valve disease in people aged 70–89 years is 20–50 times higher than in people aged 50–59 years (5). In addition, approximately 24.2 million people worldwide suffer from mitral regurgitation (MR), a common type of VHD. Over the past 30 years, the prevalence of MR has increased by about 70% mainly in many developing countries (6). Tricuspid valve disease (TVD) is less frequently reported, but in actual clinical practice, its prevalence is unlikely to be low, and atrial fibrillation, along with population aging and increased pacemaker implantation, does seem to partially explain the current increased burden of TVD (7, 8).

The Global Burden of Diseases (GBD) project is an international research project that spans multiple countries and regions and aims to assess the burden of disease in countries and regions around the world. The GBD database is maintained by the Institute for Health Metrics and Evaluation (IHME) and is fully open to the public. At present, there are several studies on calcific aortic valve disease burden using GBD data (9–13). Overall, most of these studies have shown that incident, prevalent, and death cases, and disability-adjusted life years (DALYs) as well as the incidence, prevalence, mortality, and DALYs rate of CAVD worldwide from 1990 to 2019. These findings demonstrated age-standardized mortality rate (ASMR) has remained relatively stable, and the age-standardized DALYs rate (ASDR) for CAVD has declined over the past 30 years. In addition, CAVD is also most heavily burdened in high SDI regions.

Recently, several studies have explored the disease burden of NRVHD. For example, one study assessed the burden of NRVHD in China and predicted the burden over the next 25 years (14). Another study assessed and compared the quality and equity of nursing care for NRVHDs in different SDI regions (15). In other GBD studies, the authors evaluated the incidence and mortality of VHD based on the GBD 2017 (16, 17). In the current study, we comprehensively explored the burden of NRVHD using GBD 2019 data, including: (1) investigating the age-standardized incidence rate (ASIR), age-standardized prevalence rate (ASPR), ASMR, and ASDR of NRVHD from 1990 to 2019; (2) exploring the global trends of the burden of NRVHD disease; (3) assessing possible risk factors for NRVHD; (4) predicting the disease burden of NRVHD over the next 15 years.

## 2 Methods

### 2.1 Data source

In the current study, we obtained the crude and age-standardized primary measures about NRVHDs from 1990 to 2019 from the GBD 2019 data in September 2023, which was designed to provide a comprehensive assessment of health loss due to diseases, causes of death, and risk factors at the global, regional, and national levels from 1990 to 2019 (18, 19). The GBD network employs a standardized Bayesian framework to generate disease estimates using all existing data across age, gender, time, and geographic region, and across different health causes and domains, which enables information to be gleaned from existing data to estimate the global disease burden for the vast majority of countries and regions (20).

In our study, NRVHD was defined based on the International Classification of Diseases Ninth Revision (ICD-9) and Tenth Revision (ICD-10) codes. In GBD 2019, a new disease modeling tool named DisMod-MR-2.1 was employed to provide a comprehensive summary rating of the disease burden of NRVHD by gender, age, and SDI for 204 countries or territories worldwide from 1990 to 2019 (20). Mortality data are derived from registration data defined by the International Classification of Diseases (ICD) code. DALYs due to NRVHD refer to the healthy life year loss from illness to death, which is the sum of years of life lost and years lived with disability. In addition, the age-standardized rates (ASRs) of prevalence, incidence, mortality, and DALYs were generated by summarizing the products of the age-specific rates and corresponding number of persons in the same age subgroup of the GBD 2019 standard population, and then dividing by the sum of the standard population weights (1, 2). This study is performed according to the Guidelines for Accurate and Transparent Health Estimates Reporting.

### 2.2 Risk factor analysis

GBD 2019 assessed risk factors in 204 countries and territories around the world, and derived a total of four levels of risk factors, which were divided into 3 grade 1 groups, 20 grade 2 groups, 52 level 3 groups, and 69 level 4 groups. In this study, we estimated the attributable mortality and DALYs for 87 risk factors that may be associated with NRVHD. Definitions of all included risk factors are available on the Global Health Data Exchange website.

### 2.3 Statistical analysis risk factor analysis

First, we analyzed the global disease burden of NRVHD in 1990 and 2019 and further performed subgroup analyses according to age, sex, and SDI region. Second, we also analyzed trends in ASIR, ASPR, ASMR, and ASDR of NRVHD, at the same time, trends of incidence, prevalence, mortality, and DALY rates were also assessed by sex and age groups. In addition, attributable risk factors of NRVHD for DALYs and mortality from 1990 to 2019 were analyzed using GBD 2019 data. Finally, the Nordpred age-period-cohort model is also employed to predict the disease burden of NRVHD from 2020 to 2035.

In the study, the Joinpoint regression model was used to analyze the trends of prevalence, incidence, mortality, and DALY rates. The Joinpoint regression model is a set of linear models, and the joint points of adjacent linear models are inflection points (21). By identifying the position, number and *p* value of inflection points, the statistical significance of each linear model in the set is analyzed, to judge the trend of epidemiological characteristics over time. The Joinpoint regression model was used to calculate the annual percentage change (APC) and average annual percentage rate change (AAPC). If the confidence interval (CI) of APC contains zero, it indicates that the change trend of the linear model in this segment is not statistically significant. If the APC of the segment > 0, the rate of disease is increasing year by year, and if the APC of the segment is <0, the rate of disease is decreasing year by year. If there is no inflection point throughout the process, then APC = AAPC is displayed, indicating that the rate of disease is monotonically decreasing or increasing. *P* less than 0.05 indicates that the difference is statistically significant.

We used Nordpred age-period-cohort analysis to predict the incident, prevalent, death cases, DALYs, and ASR of NRVHD from 2020 to 2035, which was implemented in R software by the package NORDPRED using a power function that grows smoothly, predicting three to four five-year observation periods. Nordpred is a well-established software tool that models count data using a Poisson distribution and employs either a log-link or a power-link function with a fixed power (22). In this model, the number of new cases projected in 2035 is a weighted average of the incidence cases through the last two periods centered on 2035.

## 3 Results

### 3.1 Global ASIR and AAPC of non-rheumatic valvular heart disease

The global incidence of NRVHD in 2019 was 1,653,553,000, with an ASIR of 19.77 per 100,000 people, and its AAPC value was 0.27(95% CI 0.19–0.34), suggesting a high global incidence (Table 1).

**Table 1:** NRVHD cases, age-standardized incidence rates, and age-standardized average annual percent change, from 1999 to 2019.

Characteristics	1990		2019		1990–2019		
	Cases (95% *UI*)	ASR (per 100 000) (95% *UI*)	Cases (95% *UI*)	ASR (per 100 000) (95% *UI*)	AAPC (95% *CI*)	*t*-test	*P*
Global	808034.30 (768445.00–850866.20)	18.37 (17.41–19.35)	1653553.00 (1556532.00–1755010.00)	19.77 (18.62–20.95)	0.27 (0.19–0.34)	6.65	<0.001
Sex							
Male	335695.88 (317867.50–354329.02)	15.61 (14.77 −16.51)	755572.38 (704035.62–809802.59)	18.64 (17.39–19.88)	0.64 (0.55–0.73)	14.01	<0.001
Female	472338.46 (448607.06–496452.04)	21.03 (19.97–22.15)	897980.89 (847500.10–950572.00)	20.83 (19.68–22.02)	−0.02 (−0.12–0.08)	−0.38	0.704
Age							
<20 years	6683.67 (6171.22–7268.60)*	0.29 (0.27–0.32)*	6814.66 (6270.41–7422.39)*	0.25 (0.23–0.27)*	−0.55 (−0.57–0.52)	−40.41	<0.001
20–54 years	377828.80 (331905.70–430189.50)*	17.14 (14.96–19.62)*	597428.00 (520293.10–685680.00)*	15.42 (13.45–17.67)*	−0.38 (−0.46–0.30)	−9.53	<0.001
55–94 years	423077.80 (352981.40–498722.40)*	61.09 (50.72–72.35)*	1044799.00 (872879.00–1243370.00)*	73.94 (61.61–88.21)*	0.71 (0.56–0.87)	8.97	<0.001

* Calculated 95% CI.

NRVHD, non-rheumatic valvular heart disease; ASR, age-standardized rate; AAPC, average annual percentage change; UI, uncertain interval; and CI, confidence interval.

In the subgroup analysis by sex, the changes in ASIR globally are predominantly male, from 15.61 (95% UI 14.77–16.51) per 100,000 people in 1990 to 18.64 (95% UI 17.39–19.88) per 100,000 people in 2019, with an AAPC of 0.64 (95% CI 0.55–0.73), while there was a downward trend of the ASIR in females. In the subgroup analysis by age, compared to 1990, the ASIR of NRVHD decreased in people younger than 54 years; However, in people aged 55–94 years, the ASIR value of 73.94 (95% UI 61.61–88.21) per 100,000 people increased significantly, and its AAPC was 0.71 (95% CI: 0.56–0.87) (Table 1). In subgroup analysis with SDI, ASIR was significantly increased in all countries and regions, especially in high SDI (56.45 per 100,000 people, 95% UI 53.35–59.83) and high-middle SDI region (27.68 per 100,000 people, 95% UI 25.91–29.57); and middle SDI countries had the largest AAPC with a value of 1.13 (95% CI: 1.09–1.17) (Table 1). When subgroup analysis is done by location, ASIR increased in all regions except Central Sub-Saharan Africa and Eastern Sub-Saharan Africa (Supplementary Table S1). In 2019, the regions with the highest ASIR were, in descending order, high-income North America (80.10 per 100,000 people, 95% UI 76.39–84.01), high-income Asia Pacific (75.40 per 100,000 people, 95% UI 70.66–80.50), Central Europe (67.65 per 100,000 people, 95% UI 61.51–74.68) and Australasia (57.00 per 100,000 people, 95% UI 50.39–64.65). The AAPC in descending order was Andean Latin America (3.98, 95% CI: 3.75–4.21), Southern Sub-Saharan Africa (3.83, 95% CI: 3.71–3.96), Australasia (3.78, 95% CI: 3.72–3.85), and Southern Latin America (2.67, 95% CI: 2.61–2.73) (Supplementary Table S1).

### 3.2 Global ASPR and AAPC of non-rheumatic valvular heart disease

The number of NRVHD patients worldwide in 2019 was 32,598,380 (95% UI 30,855,100–34,336,340). The ASPR was 99.5 (95% UI 378.31–420.75) per 100,000 people, and its AAPC value was 0.09 (95% CI: 0.04–0.14), indicating a mild increase (Table 2).

**Table 2:** NRVHD cases, age-standardized prevalence rates, and age-standardized average annual percent change, from 1999 to 2019.

Characteristics	1990		2019		1990–2019		
	Cases (95% *UI*)	ASR (per 100 000) (95% *UI*)	Cases (95% *UI*)	ASR (per 100 000) (95% *UI*)	AAPC (95% *CI*)	*t*-test	*P*
Global	15,52,5350 (14,78,5330–16,27,9190)	391.40 (372.71–411.20)	32,59,8380 (30,85,5100–34,33,6340)	99.50 (378.31–420.75)	0.09 (0.04–0.14)	3.56	0.001
Sex							
Male	59,65,561 (56,55,844–62,78,101)	321.67 (305.05–339.23)	13,97,4790 (13,09,2370–14,86,5040)	364.63 (341.91–387.31)	0.46 (0.38 to 0.53)	12.12	<0.001
Female	95,59,785 (91,19,797–10,02,9440)	448.40 (427.42–470.25)	18,62,3590 (17,72,5740–19,55,1360)	427.24 (406.78–448.79)	−0.17 (−0.23–0.12)	−5.74	<0.001
Age							
<20 years	5762.36 (4965.98–6581.18)*	0.25 (0.22–0.29)*	5460.87 (4726.40–6219.25)*	0.20 (0.17–0.23)*	−0.79 (−0.91–0.67)	−13.07	<0.001
20–54 years	4324279.00 (40,60,350–46,30,249)*	202.57 (186.55–221.16)*	66,72,388 (62,19,668–71,64,128)*	170.89 (156.83–186.76)*	−0.59 (−0.63–0.55)	−30.35	<0.001
55+ years	11,19,5300 (10,58,0520–11,85,2520)*	11745.99 (1630.99–1864.26)*	25,92,0530 (24,44,0370–27,51,4620)*	1876.01 (1744.07–2016.97)*	0.25 (0.15–0.35)	5.09	<0.001

* Calculated 95% CI.

NRVHD, non-rheumatic valvular heart disease; ASR, age-standardized rate; AAPC, average annual percentage change; UI, uncertain interval; and CI, confidence interval.

In subgroup analysis by sex, similar to the results of ASIR, the changes in global ASPR were also predominantly male, from 321.67 (95% UI 305.05–339.23) per 100,000 people in 1990 to 364.63 (95% UI 341.91–387.31) per 100,000 people in 2019, with an AAPC of 0.46 (95% CI: 0.38–0.53), while the ASPR of female showed a downward trend. In the subgroup analysis by age, compared to 1990, the ASPR of NRVHD decreased in people younger than 54 years (Table 2); However, in people aged 55–94 years, the ASPR value of 1,876.01 (95% UI 1,744.07–2,016.97) per 100,000 people increased significantly, and its AAPC was 0.25 (95% CI: 0.15–0.35) (Table 2). For SDI, ASPR was significantly increased in all countries and regions, especially in high SDI; and the low and low-middle SDI regions had the largest AAPC, with values of 1.1(95% CI: 1.07–1.13) and 0.64 (95% CI: 0.58–0.69), respectively. When subgroup analysis is done by location, ASPR increases in all regions except Central Sub-Saharan Africa and Eastern Sub-Saharan Africa (Supplementary Table S2). In 2019, the regions with the highest ASPR were High-income Asia Pacific (1,553.60 per 100,000 people), High-income North America (1,438.37, per 100,000 people), and Central Europe (1,292.65 per 100,000 people). The AAPC in descending order is Andean Latin America, Southern Sub-Saharan Africa, Caribbean, Australasia, Central Latin America, and Southern Latin America (Supplementary Table S2).

### 3.3 Global ASMR and AAPC of non-rheumatic valvular heart disease

The global number of deaths in 2019 was 164,124.60 (95% UI 140,082.20–179,557.60) for NRVHD. The ASMR was 2.25 (95% CI: 1.89–2.47) per 100,000 people, and its AAPC value was −0.36 (95% UI −0.45 to −0.26), indicating a reduction in global mortality (Table 3).

**Table 3:** NRVHD cases, age-standardized mortality rates, and age-standardized average annual percent change, from 1999 to 2019.

Characteristics	1990		2019		1990–2019		
	Cases (95%*UI*)	ASR (per 100 000) (95% *UI*)	Cases (95% *UI*)	ASR (per 100 000) (95% *UI*)	AAPC (95% *CI*)	*t*-test	*P*
Global	77932.19 (70797.70–83257.63)	2.48 (2.21–2.64)	164124.60 (140082.20–179557.60)	2.25 (1.89–2.47)	−0.36 (−0.45−0.26)	−7.09	<0.001
Sex							
Male	33067.72 (30563.61–35676.47)	2.43 (2.24–2.61)	68112.41 (61050.74–73325.89)	2.27 (1.98–2.45)	−0.25 (−0.34–0.17)	−5.76	<0.001
Female	44864.47 (39653.32–49052.70)	2.45 (2.15–2.66)	96012.16 (78368.37–108436.70)	2.19 (1.79–2.48)	−0.38 (−0.47–0.30)	−8.52	<0.001
Age							
<20 years	556.66 (411.58–733.22)*	0.11 (0.08–0.14)*	577.71 (462.11–713.33)*	0.09 (0.07–0.12)*	/	/	/
20–54 years	8665.25 (7272.22–10318.50)*	0.40 (0.33–0.47)*	11400.87 (9935.63–13185.47)*	0.30 (0.26–0.34)*	−1 (−1.07–0.94)	−29.53	<0.001
55–94 years	66588.86 (59633.46–71688.49)*	12.56 (11.12–13.56)*	136128.10 (115218.80–150320.50)*	10.82 (9.10–11.97)*	−0.52 (−0.6–0.43)	−11.73	<0.001

* Calculated 95% CI.

NRVHD, non-rheumatic valvular heart disease; ASR, age-standardized rate; AAPC, average annual percentage change; UI, uncertain interval; and CI, confidence interval.

In subgroup analyses by sex, we found a significant reduction in ASMR from 1990 to 2019 in both men and women, especially in women, with an AAPC of −0.38 (95% UI −0.47 to −0.3). In subgroup analysis by age, compared with 1990, the ASMR of NRVHD decreased significantly in people aged 20–54, and its AAPC is −1 (95% CI: −1.07 to −0.94) (Table 3). In addition, ASMR decreased significantly in all regions except High-middle SDI, but remained high in high SDI (3.99 per 100,000 people, 95% UI 3.31–4.38). The decline was most pronounced in the Middle SDI and High SDI, with AAPC values of −0.61(95% CI: −0.68 to −0.54) and −0.44(95% CI: −0.57 to −0.32), respectively, while the ASMR in the High-Middle SDI increased to some extent (Supplementary Table S2). Furthermore, the burden of ASMR in Western Europe (4.82 per 100,000 people, 95% UI 4.08–5.29), High-income North America (4.25 per 100,000 people, 95% UI 3.59–4.67), and Australasia 3.85 (95% UI 3.20–4.29) per 100,000 people remains high, although ASMR in these regions is mostly declining. At the same time, the most significant ASMR increases were observed in Central Asia, Central Europe, and Eastern Europe (Supplementary Table S3).

### 3.4 Global ASDR and AAPC of non-rheumatic valvular heart disease

The global DALYs of NRVHD in 2019 was 2,793,750.00 (2,518,737.00–3,129,035.00). The ASDR was 35.94 (95% UI 32.32–40.19) per 100,000 people, and its AAPC value was −0.73 (95% CI: −0.78 to −0.68), indicating a reduced disease burden globally (Table 4).

**Table 4:** NRVHD cases, age-standardized DALYs rates, and age-standardized average annual percent change, from 1999 to 2019.

Characteristics	1990		2019		1990–2019		
	Cases (95%*UI*)	ASR (per 100 000) (95% *UI*)	Cases (95% *UI*)	ASR (per 100 000) (95% *UI*)	AAPC (95% *CI*)	*t*-test	*P*
Global	1666706.00 (1490486.00–1849033.00)	44.46 (39.95–49.18)	2793750.00 (2518737.00–3129035.00)	35.94 (32.32–40.19)	−0.73 (−0.78–0.68)	−26.70	<0.001
Sex							
Male	793697.40 (727287.00–883520.30)	45.74 (42.13–50.32)	1328407.00 (1219314.00–1464183.00)	37.81 (34.64–41.78)	−0.65 (−0.70–0.60)	−25.61	<0.001
Female	873008.40 (746781.60–1000072.00)	42.44 (36.69–48.04)	1465343.00 (1274135.00–1675168.00)	33.79 (29.34–38.64)	−0.8 (−0.86–0.74)	−26.74	<0.001
Age							
<20 years	39814.95 (29447.00–52443.09)*	1.73 (1.28–2.28)*	41310.40 (33030.26–50980.33)*	1.51 (1.21–1.86)*	−0.49 (−0.66–0.32)	−5.61	<0.001
20–54 years	421963.70 (352453.40–505605.00)*	18.84 (15.82–22.45)*	546083.70 (474013.90–634340.50)*	14.25 (12.36–16.57)*	−0.96 (−1.02–0.89)	−28.66	<0.001
55–94 years	1192046.00 (1070558.00–1325313.00)*	201.89 (180.52–224.37)*	2114896.00 (1851264.00–2399907.00)*	161.10 (140.45–182.75)*	−0.78 (−0.84–0.72)	−24.08	<0.001

* Calculated 95% CI.

NRVHD, non-rheumatic valvular heart disease; ASR, age-standardized rate; AAPC, average annual percentage change; UI, uncertain interval; and CI, confidence interval.

In subgroup analysis by sex, we found that ASDR was significantly reduced from 1990 to 2019 in both men and women, especially in men, with AAPC of −0.65 (95% CI: −0.70 to −0.60). We also found that the ASDR of NRVHD decreased significantly in all populations, especially in people aged 20–54 with an AAPC of −0.96 (95% CI: −1.02 to −0.89) (Table 4). Additionally, the ASDR decreased significantly in all countries and regions, but the ASDR remained high in the high SDI region; the most pronounced decreases were observed in the high and middle SDI regions, with the AAPC values of −0.93 (95% CI: −1.05 to −0.81) and −0.63 (95% CI: −0.69 to −0.58) (Supplementary Table S4). The burden of ASDR in Western Europe, High-income North America, Central Europe, and Australasia remains high, although ASMR in these regions is also declining. The regions with the most pronounced downward trends include the High-income Asia Pacific −1.76 (95% CI: −1.92 to −1.60) and East Asia −1.32 (95% CI: −1.41 to −1.23) regions. At the same time, the most significant increases of ASDR for DALYs were also in Eastern Europe 2.10 (95% UI 1.55–2.65) per 100,000 people, Central Asia 1.95 (95% UI 1.60–2.30) per 100,000 people, and Central Europe 1.63 (95% UI 1.50–1.76) per 100,000 people. Overall, the trends in ASDR were remarkably similar to those of ASDR (Supplementary Table S4).

### 3.5 Trends of ASIR, ASPR, ASMR, and ASDR of NRVHD globally from 1990 to 2019

**Figure 1:**
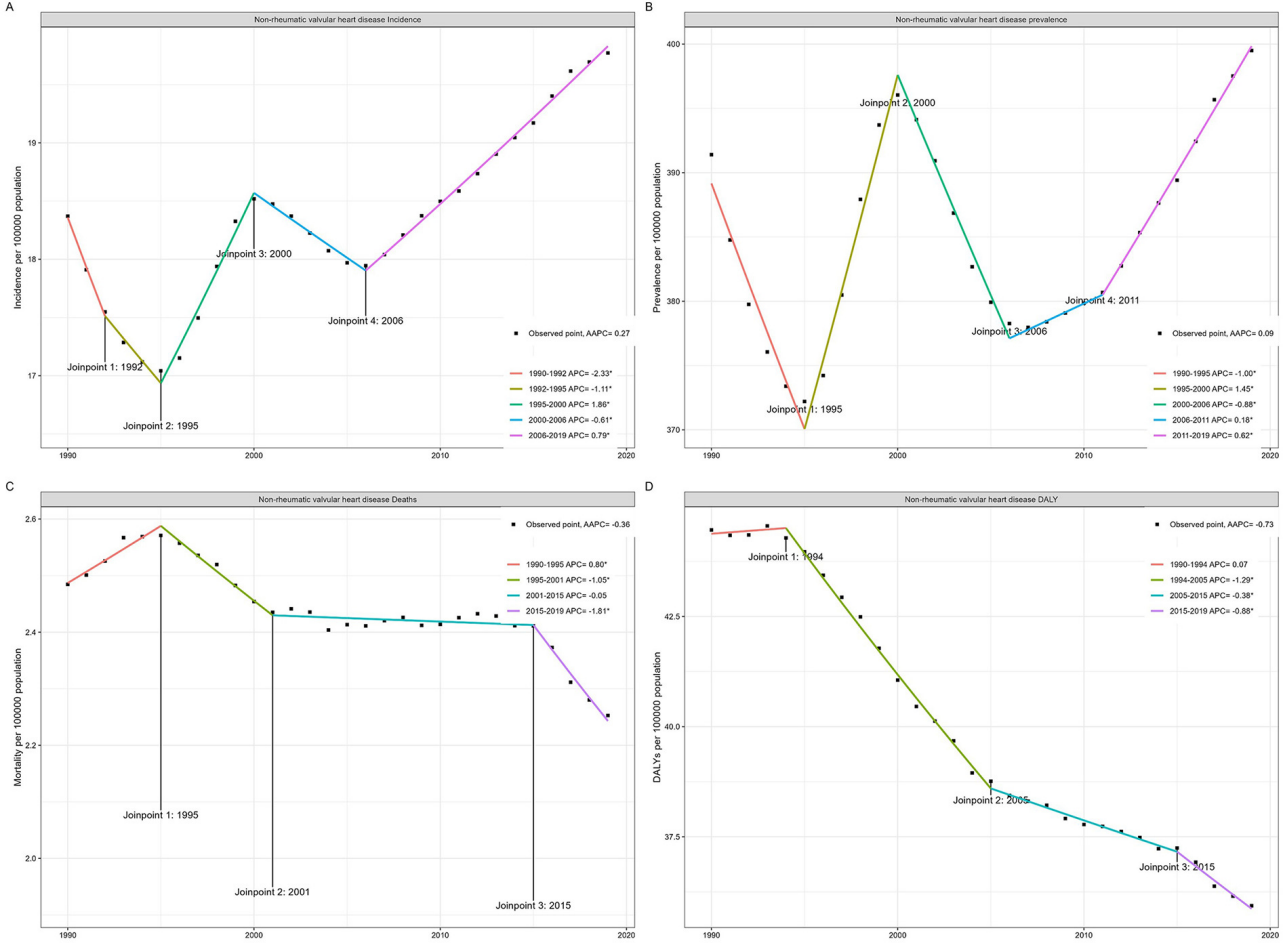
Joinpoint regression analysis of global NRVHD incidence **(A)**, prevalence **(B)**, mortality **(C)**, and DALYs **(D)**.

Overall, ASIR (AAPC = 0.27, *p* < 0.001) and ASPR (AAPC = 0.09, *p* < 0.001) of NRVHD showed an upward trend according to the Joinpoint analysis, the ASMR (AAPC = −0.36, *p* < 0.001) and ASDR (AAPC = −0.73, *p* < 0.001) showed a decreasing trend. In terms of prevalence, the ASR first decreased then rose from 1990 to 2019, and it increased most significantly from 1995 to 2000. The trend of ASIR is similar to that of prevalence (Figure 1).

**Figure 2:**
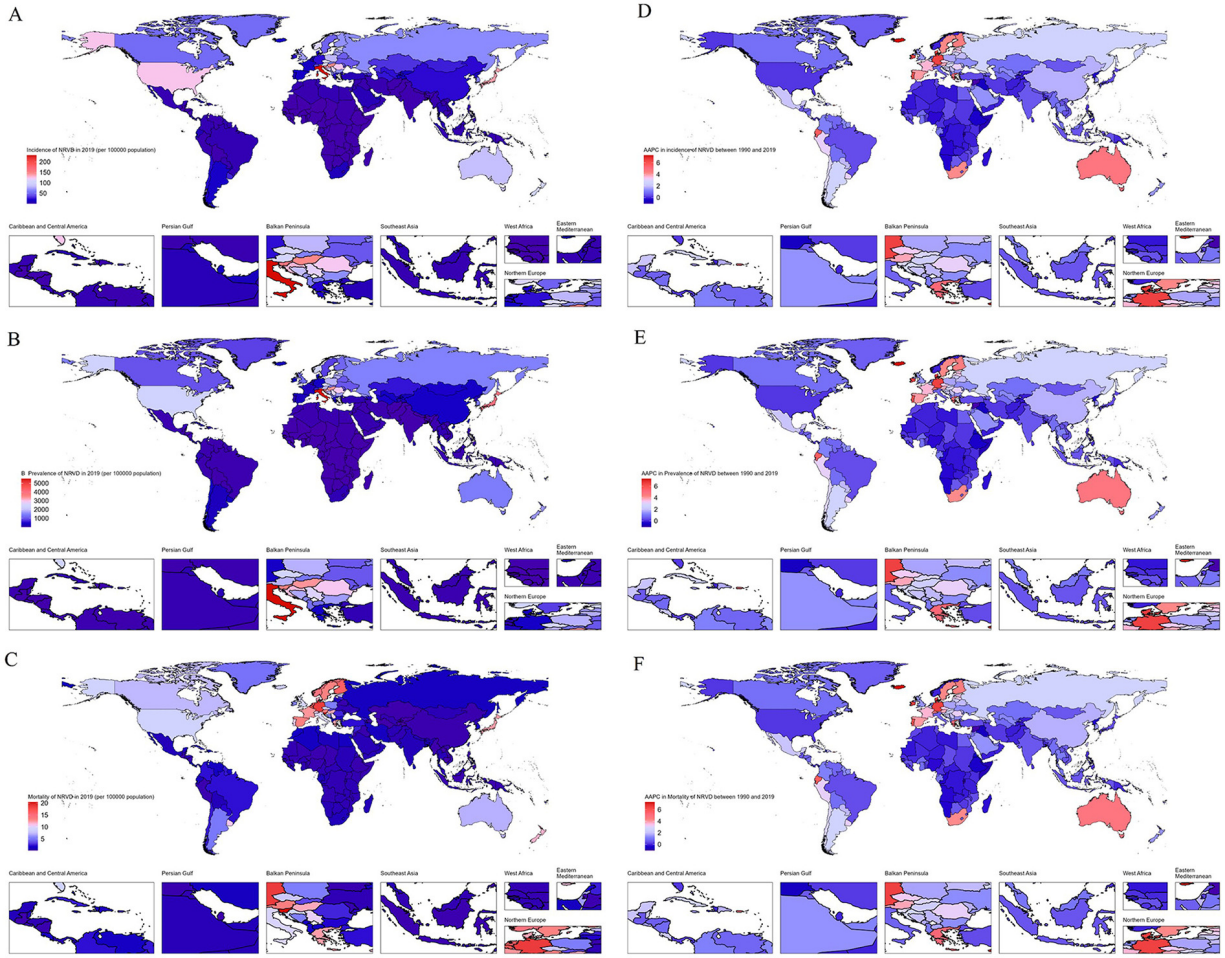
Global map of 2019 incidence **(A)**, prevalence **(B)**, mortality **(C)** of NRVHD and average annual percentage changes of incidence **(D)**, prevalence **(E)**, mortality **(F)** from 1990 to 2019.

In terms of mortality, since 1994, ASMR has been declining continuously, with the most significant declines from 2015 to 2019 (−1.81, 95% CI: −2.3 to −1.31) and from 1995 to 2001 (−1.04, 95% CI: −1.31 to −0.78). It is worth mentioning that the trend of ASDR is similar to that of prevalence. All results are detailed in Table 5 and Figure 1. A global map of AAPC in incidence, prevalence, and mortality from 1990 to 2019 is shown in Figure 2.

**Table 5:** Joinpoint trend analysis of age-standardized incidence, prevalence, mortality, DALYs rates, from 1990 to 2019.

Year	APC (95%*CI*)	*T*-test	*P*
Incidence			
1990–1992	−2.33 (−2.9 to −1.76)	−8.62	<0.001
1992–1995	−1.11 (−1.68 to −0.54)	−4.11	0.001
1995–2000	1.86 (1.67 to 2.05)	20.92	<0.001
2000–2006	−0.61 (−0.74 to −0.47)	−9.51	<0.001
2006–2019	0.79 (0.76 to 0.82)	52.43	<0.001
Prevalence			
1990–1995	−1 (−1.12 to −0.88)	−17.55	<0.001
1995–2000	1.45 (1.27 to 1.62)	17.53	<0.001
2000–2006	−0.88 (−1 to −0.76)	−15.13	<0.001
2006–2011	0.18 (0.01 to 0.35)	2.28	0.037
2011–2019	0.62 (0.56 to 0.68)	21.43	<0.001
Mortality			
1990–1995	0.8 (0.55 to 1.05)	6.67	<0.001
1995–2001	−1.04 (−1.31 to −0.78)	−8.17	<0.001
2001–2015	−0.05 (−0.12 to 0.02)	−1.44	0.166
2015–2019	−1.81 (−2.3 to −1.31)	−7.58	<0.001
DALYs			
1990–1994	0.07 (−0.16 to 0.31)	0.64	0.528
1994–2005	−1.29 (−1.34 to −1.23)	−47.49	<0.001
2005–2015	−0.38 (−0.45 to −0.31)	−11.33	<0.001
2015–2019	−0.88 (−1.13 to −0.63)	−7.36	<0.001

DALYs, disability-adjusted life years; APC, annual percentage change; CI, confidence interval.

### 3.6 Trends of ASIR, ASPR, ASMR, and ASDR of NRVHD by age and sex

The prevalence, incidence, mortality, and DALYs of NRVHD were lower in people younger than 20 years of age. Surprisingly, the disease burden of the High SDI region was also relatively small in this population, which may suggest that the disease burden of NRVHD was closely related to the regional economic level. Among the 20- to 54-year-old population, the mortality rate in the high SDI and high-middle SDI regions showed a downward trend, especially in the former. In addition, the mortality and DALY rates in the low-middle SDI region were also declining, but they were higher than those in the high SDI region in 2019. Among people aged 55–94 years, the prevalence, morbidity, and mortality rates in high SDI regions were on the rise, and DALYs were declining, but still higher than the global level.

**Figure 3:**
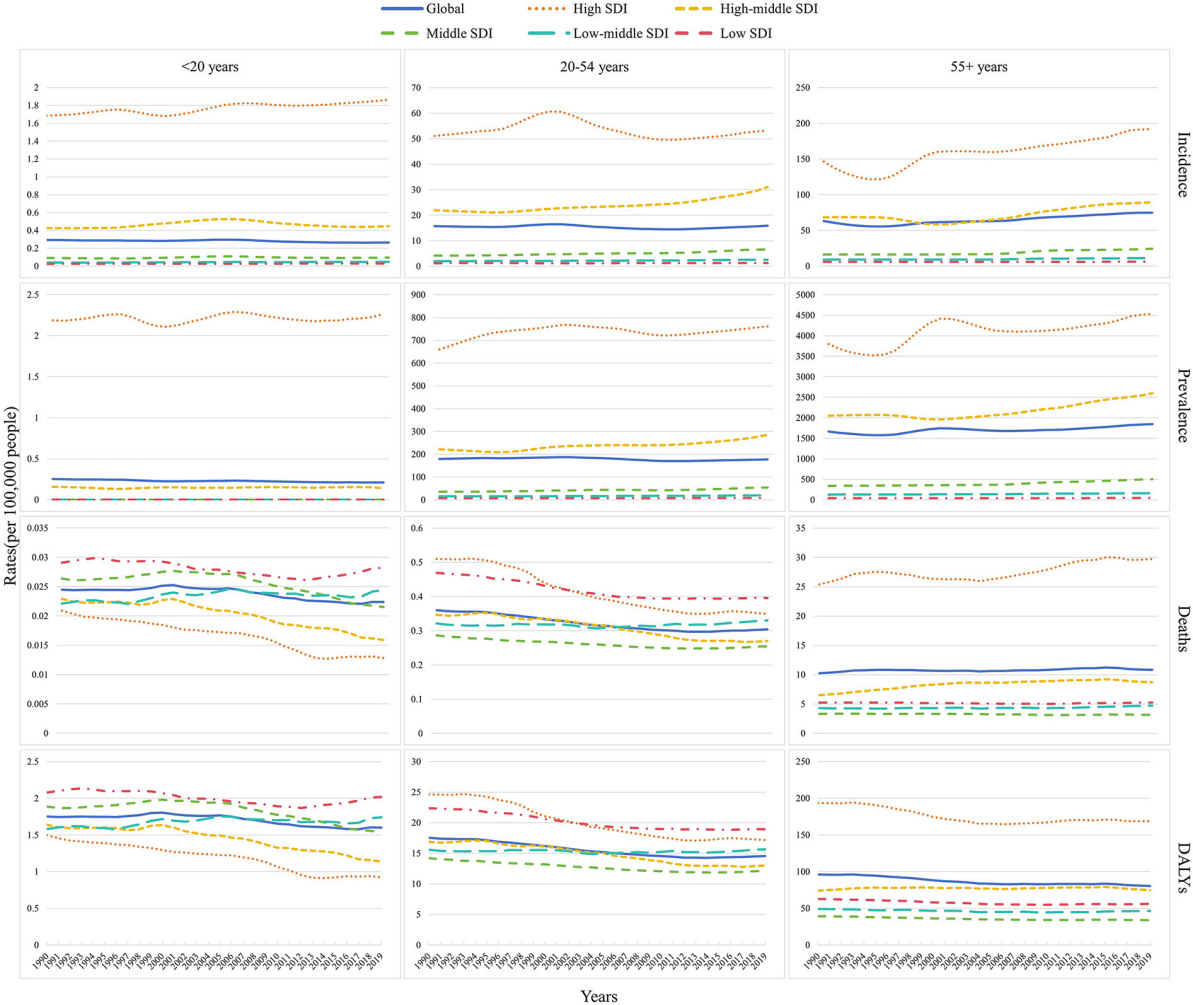
Temporal trends id incidence, prevalence, mortality, and DALYs rate of NRVHD by age (<20 years, 20-54 years, 55+ years) and sociodemographic index (high-income, high-middle income, middle income, low-middle income, and low-income categories) from 1990 to 2019.

**Figure 4:**
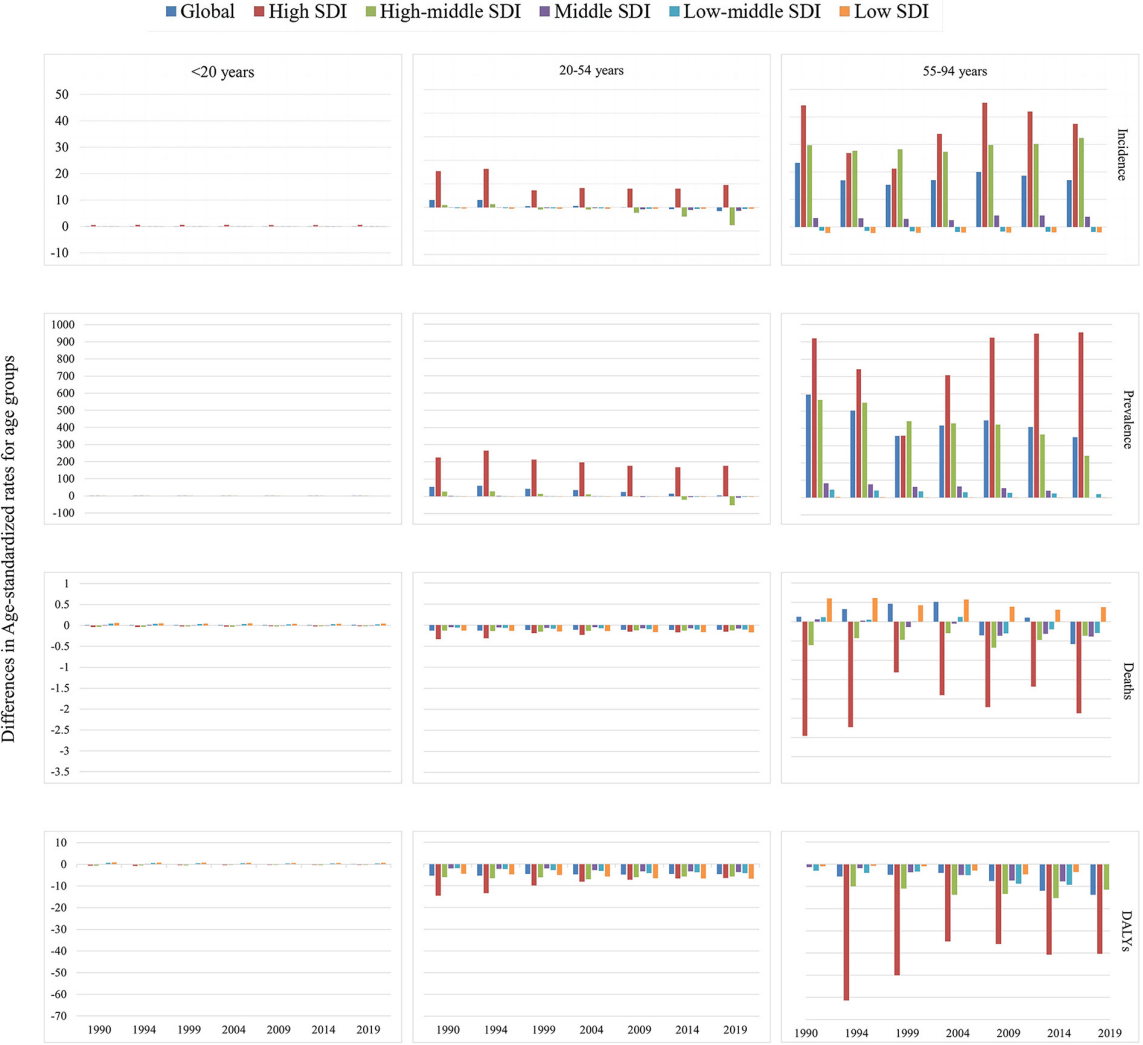
Diference in age-standardized incidence, prevalence, DALY, and mortality rate of NRVHD between men and women by age and sociodemographic index (high-income, high-middle income, middle income, low-middle income, and low-income categories), from 1990 to 2019. The diference indicates the age-standardized rate in women minus that in men. A diference > 0 suggests that women have a higher rate than men.

**Figure 5:**
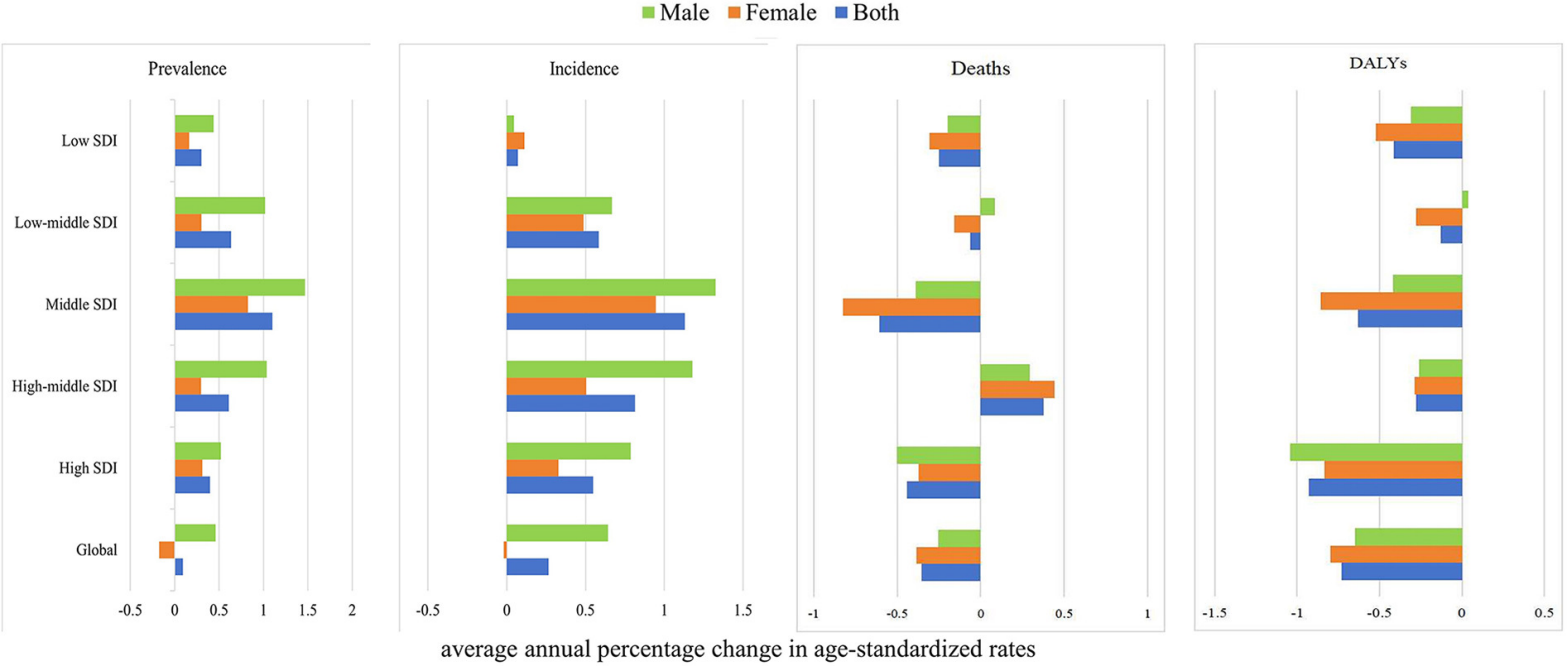
Average annual percent change in age-standardized prevalence, incidence, mortality, and DALYs rate of NRVHD by sex (men and women) and sociodemographic index (high-income, high-middle income, middle income, low-middle income, and low-income categories) from 1990 to 2019.

The global prevalence and incidence of NRVHD were increasing, especially for males in High-middle and middle SDI regions, while the prevalence and incidence of NRVHD were decreasing in females. In addition, ASMR and DALYs for males in the Low-middle SDI region, and ASMR for males and females in the High-middle SDI region, were still on the rise. Happily, DALYs overall remained on a downward trend across the all-SDI regions for both males and females. The detailed information is presented in Figures 3–5.

### 3.7 Risk factor analysis

High systolic blood pressure (SBP), high sodium diet, and lead exposure were the main attributable risk factors of mortality and DALYs for NRVHD in 2019, with the former being the most important risk factor for NRVHD. The ASMR attributable to high SBP in 2019 was 0.54

(95% UI 0.37–0.76) per 100,000 people, much higher than the high-sodium diet (0.06 per 100,000 people, 95% UI 0.01–0.15) and lead exposure (0.03 per 100,000 people, 95% UI 0.01–0.05); on the other hand, the ASDR attributable to high SBP in 2019 was 8.03 (95% UI 6.07–10.21) per 100,000 people, much higher than the high-sodium diet (0.98 per 100,000 people, 95% UI 0.20–2.44) and lead exposure (0.40 per 100,000 people, 95% UI 0.18–0.68) (Supplementary Table S5).

**Figure 6:**
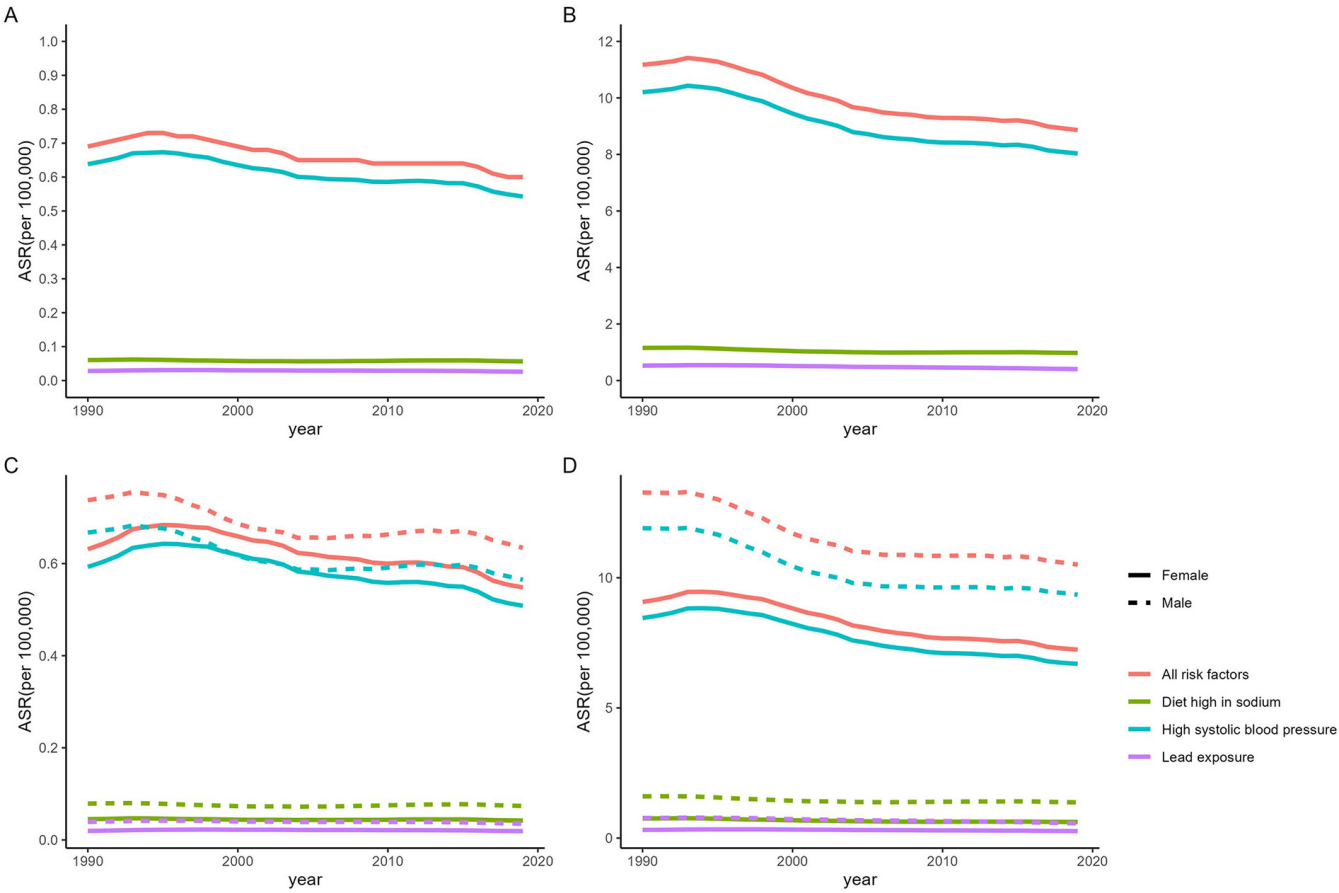
Age-standardized mortality **(A)**, DALYs **(B)** rates and Age-standardized mortality **(C)**, DALYs **(D)** rates group by sex variation of global NRVHD risk fators from 1990 to 2019.

In subgroup analysis by sex, mortality attributable to high SBP in 2019 was lower in females than in males (0.59 vs. 0.67 per 100,000 people), DALYs was also lower in females than in males (8.46 vs. 11.91, per 100,000 people). In addition, the ASMR attributable to high SBP in 2019 was higher in people over 55 years of age than in the 20-to 54-year-olds (1.46 > 0.07 per 100,000 people). Similarly, the ASDR attributed to high SBP was much higher in people over 55 years of age than in the 20–54 years (23.80 vs. 3.16 per 100,000 people). Additionally, the ASMR and the ASDR attributed to high SBP were lower in 2019 than in 1990 (0.54 vs. 0.37; 8.03 vs. 7.00 per 100,000 people). The detailed information is presented in Figure 6, Supplementary Tables S6–S9, and Supplementary Figures S1, S2.

### 3.8 Projections to 2035

**Figure 7:**
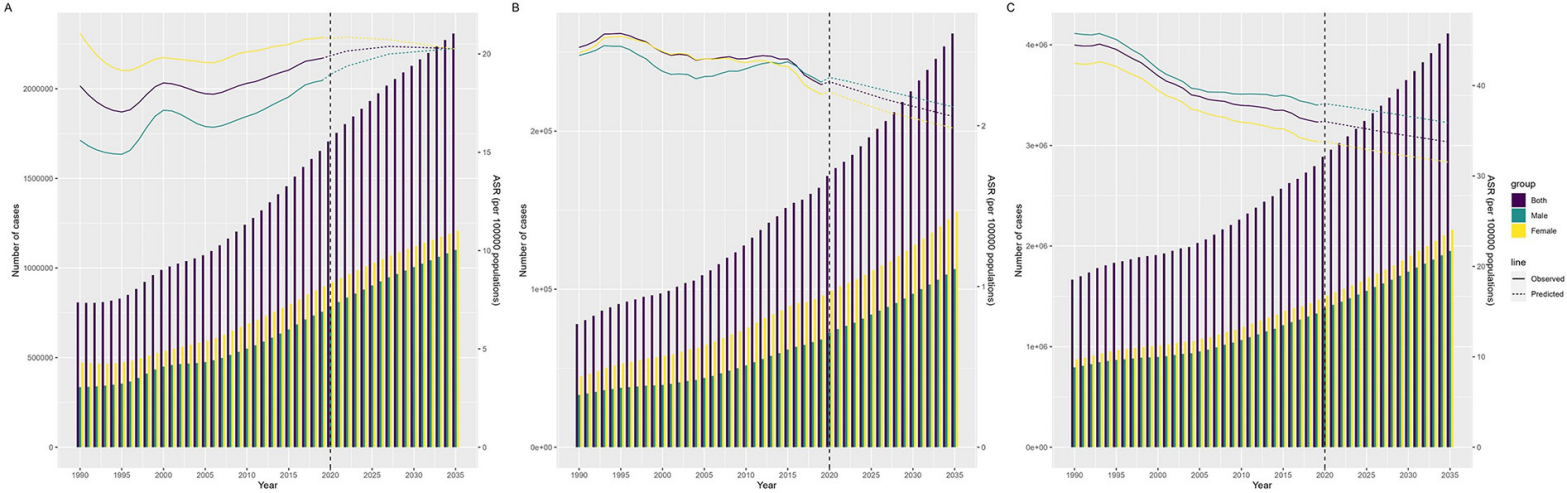
Nordpred projections of incidence **(A)**, mortality **(B)**, DALYs **(C)** numbers and age-standardized rates of NRVHD by sex in global from 1990 to 2035.

Age-period-cohort model by NORDPRED showed an upward trend in ASIR and a downward trend in disease burden globally over the next 15 years (Figure 7). It is predicted that the ASIR of NRVHD may increase to 20.28 per 100,000 people in 2035, and there may be 2,309,174 new cases, while the ASMR may decrease to 2.06 per 100,000 people, and the number of deaths may increase to 261,819; meanwhile, ASDR may decrease to 33.74 per 100,000 people with a predicted DALYs value of 4,113,473.

Taking into account gender differences, the ASIR for men may be 20.29 per 100,000 people and there may be 1,101,296 new cases worldwide in the next 15 years, and the ASIR for women may be 20.25 per 100,000 people and there may be 1,207,878 new cases. For men, the ASMR maybe 2.11 per 100,000 people and the number of deaths may rise to 112.742; for women, the ASIR may be 1.98 per 100,000 people and the number of deaths may rise to 149,076. In addition, the ASDR for men may decrease to 35.87 per 100,000 people with a predicted DALYs of 1,951,523, and the ASDR for women may decrease to 31.52 per 100,000 people with a predicted DALYs of 2,161,949.

## 4 Discussion

Based on the GBD 2019, we comprehensively assessed the disease burden, associated risk factors of NRVHD, and predicted its disease burden over the next 15 years. The results showed the incident, prevalent, and death cases, and DALYs of NRVHD gradually increased globally from 1990 to 2019. Its ASPR and ASIR are on an upward trend, while ASMR and ASDR are on a downward trend. Specifically, ASPR and ASIR in NRVHD were consistently higher in men, but tended to decrease in women; In people older than 55 years, ASPR and ASIR continued to increase with age, with ASPR increasing most significantly in the high SDI and high-middle SDI regions; ASMR and ASDR in high-middle SDI (especially in Western Europe, high-income North America and Australasia) and high-middle SDI regions remain high, although the former was declining. Further analysis also demonstrated that the disease burden of the high SDI regions was higher than in other regions, especially in men over 55 years of age. In addition, our analysis of attributable risk factors suggested that hypertension is the most important risk factor for NRVHD, which is far more important than a high-salt diet and lead exposure. The prediction model based on NORDPRED indicates that ASIR will be on the rise in the next 15 years, while ASMR and ASDR will be on the decline.

Our study found the incident, prevalent, and death cases, and DALYs of NRVHD gradually increased globally from 1990 to 2019, but there were significant differences in the burden of NRVHD in different regions, ages, and sexes. Here, the global burden of NRVHD remains high, but there are still several interesting findings from this study. First, we found that both ASIR and ASPR, as well as ASMR and ASDR, showed a clear downward trend in women, whereas ASMR alterations were less pronounced in men than in women. Second, in people older than 55 years, ASPR and ASIR continued to increase with age, but at the same time, the decline in ASMR and ASDR was relatively flat, which suggested what we will focus on in the future. The possible reason is that this population often has multiple comorbidities, thus multidisciplinary team work must be considered in the evaluation and treatment. Third, despite an overall decline in ASMR and ASDR, the disease burden was highest in high SDI regions, suggesting that health care measures must be further strengthened in high SDI areas. Further, in serval developing countries, such as Central Sub-Saharan Africa and Eastern Sub-Saharan Africa, there may be a need to improve and strengthen national health monitoring and registration, which may make GBD data more accurate. Overall, older age and males are positively associated with the burden of NRVHD, which is consistent with previous reports.

Our findings on the epidemiological patterns and temporal trends of ASIR, ASDR, and ASMR are consistent with those reported in previous related studies (14–17). Although Wang's team (17) previous study provided a detailed description of the epidemiology of NRVHD, it relied on a single linear regression model using the estimated annual percentage change (EAPC). In contrast, our study employs the Joinpoint regression model to dynamically analyze long-term trend changes, allowing for the precise identification of critical transition periods in the epidemiology of the disease. This methodological refinement effectively overcomes the limitation of the traditional EAPC approach, which assumes a constant trend, thereby enabling the AAPC metric to more accurately reflect the temporal variations in disease burden. Notably, we further developed a predictive model based on time series analysis, integrating demographic parameters and evolving risk factor trends to provide forward-looking insights for public health decision-making. More importantly, Wang's team reported a positive correlation between the ASIR, ASMR, and ASDR of NRVHD and the SDI over the past 29 years. This overall positive association suggests that as SDI increases, the burden of NRVHD also rises. However, our study reveals that this correlation varies across age groups. For instance, among individuals under 20 years old, no significant association was observed in either high- or low-SDI regions whereas in the aged 55–94 years group, the disease burden continued to increase in high SDI regions. This indicates that the overall trend may obscure age specific differences. By incorporating stratified analysis, our study provides a more nuanced perspective on these variations.

Furthermore, in terms of study population selection, our research offers broader representativeness compared to the work of Liu's team. While Liu's team (23) also applied the Joinpoint regression method in their study, their analysis was restricted to female participants. In contrast, our study established a cohort encompassing all genders and multiple age groups. This approach ensures greater clinical relevance and enhances the generalizability of our findings to diverse populations.

With regard to relevant interventions, our study highlights the novel targeted agent ataciguat (24). The most recent findings from 2025 demonstrate that ataciguat exerts favorable therapeutic effects in valvular disease (24). This targeted approach represents a promising strategy to modify the disease process at the molecular level, potentially altering the course of disease progression. This study will be discussed in detail later in the paper.

It is worth mentioning that there have been several studies exploring the burden of CAVD through the GBD database (9–13). Currently, CAVD is the most interesting type of NRVHD, and other types of NRVHD are relatively rarely reported. Generally, the overall burden of CAVD since 1990 has been high, with the rise of ASPR, some decline in ASDR, and little change in ASMR. These findings differed results from our findings. The specific reasons may lie in the following aspects. First, we thought of NRVHD as a whole, encompassing its different types. That is, we included not only CAVD but also degenerative mitral valvular heart disease (DMVHD) and other NRVHD. Second, DMVHD had a larger population base than CAVD and was less likely to cause death. Actually, CAVD and DMVHD caused 102,700 and 35,700 deaths, and 18.1 million prevalent cases of DMVHD existed in 2017, respectively (6). A plausible explanation is that the continuous decline in ASMR of DMVHD and other NRVHD patients canceled out the stable trend of ASMR in CAVD patients, as reflected in the continued decline of ASMR for NRVHD in our study. Third, in recent decades, early intervention and the introduction of percutaneous valve therapies for patients who were previously ineligible for surgery due to operative risk and comorbidities have provided clinical benefits and improved prognosis in patients with non-rheumatic valvular heart disease (NRVHD) (25). However, comparing other NRVHD, deaths and DALYs from CAVD remained relatively severe. Fourth, Moderate/severe tricuspid regurgitation was often associated with increased all-cause mortality risk (26), but such deaths are often not attributed to deaths related to tricuspid valve disease. For example, tricuspid valve disease, especially tricuspid regurgitation, is often associated with pulmonary hypertension, atrial fibrillation, and heart failure. Yet, the cause of death in these patients is most likely attributable to pulmonary hypertension and heart failure.

Our study identified the three most important risk factors for NRVCD: high SBP, high-sodium diet, and lead exposure. Given that deaths attributed to high SBP in 2019 were more than 100 times higher than the latter two, high SBP should be our focus. High SBP is a major public health challenge that is closely linked to many chronic diseases (27). The PROGRESSA study have found that high SBP is closely related to the progression of aortic valve calcification (28), and the specific mechanism may be related to the valve interstitial cell activation, increased early diastolic tensile stress, as well as increased bending stress on the valve leaflet during the ejection stage. Further epidemiological study has found a significant increase in high SBP-related deaths and DALYs from 1990 to 2019 In contrast, ASMR and ASDR decreased by 27.0% and 27.8%, respectively (27). The trend is very similar to that of NRVHD. Globally, approximately 59% of women and 49% of men suffered from hypertension in 2019, and treatment and control rates have improved in most countries, but control rates are only around 20%, suggesting that the burden of hypertension worldwide remains high (29). Therefore, further treatment and management of hypertension is imperative. The other two main risk factors, high-sodium diet and lead exposure, have been linked to cardiovascular disease and related mortality in several studies (30, 31), which is similar to our findings. Nevertheless, direct evidence between high-sodium diets, lead exposure, and NRVHD risk has not been established, and high-quality epidemiological trials are still lacking.

Based on the current GBD data, we also predicted the global ASIR, ASMR, and ASDR of NRVHD in the next 15 years. This is also the first study to predict the global disease burden of NRVHD. The results showed an increase in the incident, prevalent, and death cases, and DALYs of NRVHD increased, but a decrease in ASMR and ASDR. Overall, this trend is similar to the epidemiological trend of NRVHD in the past 30 years, suggesting that the burden of NRVHD is still high and that appropriate public health measures are necessary. For example, we need to strengthen the prevention of NRVHD, and control potential risk factors. Specifically, smoking cessation, regular physical activity, weight management, and dietary improvements have been shown to reduce the risk of degenerative valve calcification, which may help slow the progression of NRVHD, according to the INTERHEART trial (32). A recent review on CAVD suggested that lowering plasma levels of low-density lipoprotein (LDL) and plasma lipoprotein(a) [Lp(a)] may be the most effective strategy for preventing the onset of valvular calcification (33). Routine and community-based screening plays a crucial role in the early detection of NRVHD. This underscores the critical importance of reinforcing primary prevention strategies and effectively managing modifiable risk factors to reduce the incidence and progression of valvular calcification.

We also need to popularize the screening by cardiac ultrasound (echocardiography and point-of-care ultrasound) for NRVHD. Integrating cardiac ultrasound into routine cardiovascular assessment, particularly for high-risk individuals, enables early detection of valvular dysfunction. Cardiac ultrasound is the primary modality for detecting and quantifying severity of valvular heart disease and is noninvasive and widely available (34). Additionally, community outreach programs can leverage portable diagnostic devices to screen asymptomatic individuals, facilitating the early detection of valve disease.

Timely valve intervention should be pursued in patients who are candidates for surgical or transcatheter interventions (1, 2). For patients with severe symptomatic valvular disease, conventional surgical repair or replacement remains the gold standard, offering established benefits in symptom relief, hemodynamic improvement, and long-term survival. In patients deemed inoperable, a meta-analysis (35) has shown that TAVI significantly reduces mortality. The 10-year outcomes of the NOTION trial (36) demonstrated comparable results between TAVI and surgical aortic valve replacement (SAVR) in a high-risk population.

Additionally, beyond surgical interventions, emerging therapies are being explored to reduce disease burden, the research on targeted drugs also needs to be further implemented. For instance, ataciguat has shown promise by slowing calcification progression by nearly 70% over six months in patients with moderate aortic stenosis compared to placebo and tends to slow the progression of valve and ventricular dysfunction (24). Ataciguat is an investigational drug, originally developed by Sanofi, and is currently being evaluated in a clinical trial led by the Mayo Clinic. A larger, late-stage trial is being planned by the Mayo Clinic in collaboration with industry partners. While statins have not been effective in slowing valve disease progression, the development of drugs that specifically target calcification and inflammatory pathways may provide new therapeutic options.

The number of current studies focused on predicting NRVHD remains limited. However, a Chinese study (14) on NRVHD prediction employed the same modeling approach as our study, using the Nordpred model. In that study, the model's predictions were reliably validated, supporting its applicability in this context. In another study (37) predicting the GBD in cirrhosis, the Nordpred model was used for projection, while the Bayesian Age-Period-Cohort (BAPC) model was employed for validation. The results from both models were consistent. Unlike Nordpred, the BAPC model is a stochastic framework that incorporates Bayesian statistical methods into the traditional age-period-cohort analysis. This Bayesian approach allows the model to better handle data with high uncertainty. Therefore, given the relatively stable nature of the GBD data in our study, the Nordpred model may be more appropriate for long-term forecasting.

However, Nordpred models are built on established mathematical and statistical principles; however, they may not fully capture the complex social, behavioral, and biological factors that influence disease patterns. For instance, these models may inadequately account for the impact of emerging treatments or newly introduced drugs on disease morbidity, potentially leading to discrepancies between projected and actual outcomes. To address the limitations of the Nordpred model, deep learning-based predictive models can be employed. These models are capable of automatically learning complex features and patterns from large datasets, potentially offering more accurate predictions of disease incidence trends. Additionally, a hybrid modeling approach that integrates multiple predictive models can be considered. Such an approach leverages the strengths of different methodologies, enhancing both the accuracy and reliability of forecasts.

There are still some flaws in this study. First, NRVHD data are not always available, for example, NRVHD data for people under 20 years of age are missing in the study. Especially, due to the lack of nationwide health surveillance, epidemiological data are scarce in some low and middle SDI regions, which may affect the accuracy of estimates, thus the disease burden of NRVHD may be underestimated in these countries. Second, the diagnosis of NRVHD is based on non-invasive imaging, namely cardiac ultrasound, and which may not be widely readily available in low-income countries. Third, we estimated AAPC values using Jointpoint, however, our analysis used data with a 5-year interval, which may not reflect subtle changes in AAPC; furthermore, the AAPC may also be affected by the spread of major global infectious diseases, changes in health policy, and innovative medical devices or drugs. Fourth, exclusive causes of death are primarily used in the GBD database, however, in the real world, older adults typically suffer from multiple diseases at the same time; deaths may not be only attributable to NRVHD, so their actual mortality may be underestimated. Fifth, considering the possible impact of other potential risk factors, no correlation analysis between ASMR, ASDR, and SDI was done here. Sixth, the impact of COVID-19 may have affected the accuracy of our prediction of the NRVHD burden. Seventh, Nordpred models are constructed primarily based on historical data and may exhibit some lag or lack of adaptability in response to rapidly changing environments. Lastly, due to clinician neglect, asymptomatic or mild non-rheumatic mitral and tricuspid valve disease was often underappreciated in the clinic, and therefore, these data were rarely registered in GBD, which may lead to a causal distribution bias in NRVHD.

Future research should prioritize strengthening data collaboration by establishing cross-country and cross-regional research networks to facilitate the sharing of data resources and mitigate issues related to missing data. Particular emphasis should be placed on collecting data from low-income regions and younger populations, as these groups are often underrepresented, yet critical for capturing a comprehensive picture of disease burden and the impact of interventions. While statistical methods such as multiple imputation can be employed to estimate missing values, careful consideration must be given to the validity and reliability of the interpolated data to ensure robust and accurate analyses.

In conclusion, the disease burden of NRVHD remained high globally, especially in high SDI regions; Furthermore, given the substantial burden among the elderly and the observed increase in burden within the male population, it is necessary to pay greater attention to these two groups. Priority should be given to measures such as optimizing community screening programs for the elderly and strengthening longitudinal follow-up investigations for men. It was gratifying to note that ASMR and ASDR have continued to decline globally over the past decade, particularly in regions such as East Asia and the high-income Asia Pacific. This trend was likely to continue for the next 15 years. These findings suggested that some of the current emerging medical measures including TAVI may reduce the disease burden of NRVHD; additionally, control strategies for hypertension may also play a major role in this.

## Data Availability

The datasets presented in this study can be found in online repositories. The names of the repository/repositories and accession number(s) can be found in the article/Supplementary Material.
