# Supplementary material for: Global, regional, and national burden of non-rheumatic valvular heart disease and Its projections to 2035: comprehensive analysis of the global burden of disease study 2019
Source: Front Cardiovasc Med. 2025 Aug 1;12:1445024. doi: 10.3389/fcvm.2025.1445024 (PMC12354459; doi:10.3389/fcvm.2025.1445024)
Supplement: Supplementary file 1 [file Datasheet1.pdf]

Supplementary Material

Global, Regional, and National Burden of Non-rheumatic Valvular Heart Disease and Its Projections to 2035: Comprehensive Analysis of the Global Burden of Disease Study 2019

Tao Ying[1], Qiong Nie[1], Wei Yan [1], Han Wang [2*,B], Jing Wu [1*,A]

*** Correspondence:**
Corresponding Author A: Jing Wu
wujing1@swjust.edu.cn
Corresponding Author B: Han Wang
wanghan@swjtu.edu.cn

Contents:

Supplementary Tables:

Table 1 Number of NRVHD patients, age-standardized incidence rates, and age-standardized average annual percent change, from 1999 to 2019.
Table 2 Number of NRVHD patients, age-standardized prevalence rates, and age-standardized average annual percent change, from 1999 to 2019.
Table 3 Number of NRVHD patients, age-standardized mortality rates, and age-standardized average annual percent change, from 1999 to 2019.
Table 4 Number of NRVHD NRVHD patients, age-standardized DALY rates, and age-standardized average annual percent change, from 1999 to 2019.
Table 5 Deaths and DALYs age-standardized rates of NRVHD attributable to all risk factors(diet high in sodium, lead exposure, high systolic blood pressure) in Global from 1990 to 2019.
Table 6 Deaths rates of NRVHD attributable to all risk factors(diet high in sodium, lead exposure, high systolic blood pressure) by age in Global from 1990 to 2019.
Table 7 DALY rates of NRVHD attributable to all risk factors(diet high in sodium, lead exposure, high systolic blood pressure) by age in Global from 1990 to 2019.
Table 8 Deaths age-standardized rates of NRVHD attributable to all risk factors(diet high in sodium, lead exposure, high systolic blood pressure) by sex and location in Global from 1990 to 2019.

Table 9 DALY age-standardized rates of NRVHD attributable to all risk factors(diet high in sodium, lead exposure, high systolic blood pressure) by sex and location in Global from 1990 to 2019.

Table 10 Incidence, Deaths and DALYs projections of NRVHD in Global from 2020 to 2035.

Table 11 Incidence, Deaths and DALYs projections of NRVHD by sex in Global from 2020 to 2035.

Supplementary Figures:

Supplementary Figure 1 Changes in Global NRVHD Risk Factors for Mortality (A), DALYs (B) in the 20-54 years group and Mortality (C), DALYs (D) in the 55+ years group from 1990 to 2019.

Supplementary Figure 2 Changes in Sex Differences in Mortality (A), DALYs (B) for 20-54 years group and Mortality (C), DALYs (D) for 55+ years group for global NRVHD risk factors from 1990 to 2019.

Table 1 Number of NRVHD patients, age-standardized incidence rates, and age-standardized average annual percent change, from 1999 to 2019

	1990		2019		1990-2019		
	Cases (95%*UI*)	ASR(per 100 000) (95% *UI*)	Cases (95% *UI*)	ASR(per 100 000) (95% *UI*)	AAPC (95% *CI*)	*t*	*P*
SDI							
High SDI	471651.90 (447374.40-497714.80)	48.99 (46.57-51.56)	857244.10 (804114.10-914451.00)	56.45 (53.35-59.83)	0.55 (0.41-0.69)	7.55	< 0.001
High-middle SDI	251283.00 (237918.00-265909.50)	21.82 (20.67-23.10)	549955.50 (513381.80-588205.30)	27.68 (25.91-29.57)	0.81 (0.41-0.69)	18.60	< 0.001
Middle SDI	61479.03 (57433.70-65551.79)	4.97 (4.64-5.32)	186799.90 (174142.00-200199.50)	6.87 (6.43-7.35)	1.13 (1.09-1.17)	50.72	< 0.001
Low-middle SDI	18617.42 (17082.34-20287.05)	2.79 (2.57-3.02)	47729.79 (44061.47-51548.27)	3.29 (3.04-3.54)	0.58 (0.54-0.63)	27.94	< 0.001
Low SDI	4835.42 (4372.20-5318.22)	1.96 (1.79-2.14)	11410.07 (10335.73-12540.77)	2.00 (1.82-2.19)	0.07 (0.04-0.10)	4.90	< 0.001
Location							
Central Asia	11003.08 (9130.37-13328.53)	20.22 (16.68-24.62)	25043.54 (21231.27-29515.63)	26.06 (22.11-30.72)	0.91 (0.84-0.97)	27.70	< 0.001
Central Europe	52298.27 (46930.04-59230.74)	36.41 (32.73-41.03)	112871.20 (102069.18-125792.85)	67.65 (61.51-74.68)	2.19 (2.10-2.27)	50.92	< 0.001
Eastern Europe	59351.75 (55711.42-63077.40)	22.62 (21.34-23.91)	124762.71 (110455.54-140066.77)	43.24 (38.67-47.99)	2.29 (2.16-2.41)	35.84	< 0.001
Australasia	4312.38	19.37	25292.30	57.00	3.78 (3.72-	112.15	< 0.001

	(3957.45-4728.84)	(17.81-21.28)	(22148.68-28808.61)	(50.39-64.65)	3.85)		
High-income Asia Pacific	146246.91 (138421.29-154483.20)	71.29 (67.59-75.24)	214525.29 (197272.51-232906.16)	75.40 (70.66-80.50)	0.19 (0.15-0.22)	10.88	< 0.001
High-income North America	264549.71 (251354.64-279955.13)	79.95 (76.06-84.33)	425868.48 (403332.90-450484.08)	80.10 (76.39-84.01)	0.13 (-0.13-0.39)	0.97	0.322
Southern Latin America	3401.63 (2960.90-3952.52)	7.46 (6.46-8.65)	12886.76 (11623.68-14322.16)	16.04 (14.44-17.83)	2.67 (2.61-2.73)	86.26	< 0.001
Western Europe	129048.93 (122027.73-137813.38)	26.24 (24.92-27.94)	260310.74 (240917.49-283676.20)	37.76 (35.29-40.55)	1.24 (1.06-1.41)	13.81	< 0.001
Andean Latin America	334.34 (297.75-378.84)	1.41 (1.26-1.56)	2564.98 (2257.20-2881.33)	4.31 (3.80-4.86)	3.98 (3.75-4.21)	34.18	< 0.001
Caribbean	770.15 (704.54-844.50)	2.79 (2.55-3.05)	2913.06 (2559.29-3312.69)	5.61 (4.93-6.36)	2.44 (2.24-2.65)	23.30	< 0.001
Central Latin America	2003.07 (1819.73-2204.86)	2.06 (1.88-2.25)	7986.69 (7102.91-8877.91)	3.19 (2.85-3.53)	1.56 (1.36-1.77)	15.17	< 0.001
Tropical Latin America	3588.49 (3289.79-3939.57)	3.59 (3.26-3.98)	9889.42 (8895.82-11061.50)	4.03 (3.63-4.49)	0.38 (0.36-0.41)	28.64	< 0.001
North Africa and Middle East	8708.02 (7629.64-9881.21)	4.09 (3.62-4.60)	24171.39 (21362.50-27316.04)	4.42 (3.95-4.96)	0.31 (0.15-0.46)	3.92	< 0.001
South Asia	10451.16 (9516.44-11482.90)	1.94 (1.76-2.12)	28840.76 (26358.40-31416.21)	2.11 (1.92-2.31)	0.29 (0.27-0.32)	22.14	< 0.001
East Asia	97899.56	9.34	336410.04	15.33	1.69 (1.53-	20.60	< 0.001

	(92744.48-103570.38)	(8.84-9.87)	(317994.58-354421.18)	(14.53-16.11)	1.85)		
Oceania	162.95 (137.83-193.57)	4.23 (3.65-4.97)	438.26 (371.99-512.05)	4.77 (4.14-5.53)	0.41 (0.36-0.47)	14.40	< 0.001
Southeast Asia	9450.46 (8398.10-10603.39)	3.07 (2.74-3.45)	25128.59 (22154.92-28418.45)	3.62 (3.23-4.08)	0.59 (0.54-0.64)	23.75	< 0.001
Central Sub-Saharan Africa	531.08 (465.50-604.23)	2.30 (2.07-2.56)	1240.74 (1087.85-1412.83)	2.22 (1.99-2.46)	-0.12 (-0.17--0.08)	-5.68	< 0.001
Eastern Sub-Saharan Africa	1533.57 (1390.06-1696.95)	1.98 (1.80-2.17)	3329.27 (2980.14-3697.00)	1.84 (1.68-2.03)	-0.25 (-0.29--0.21)	-12.60	< 0.001
Southern Sub-Saharan Africa	836.33 (764.32-919.37)	2.72 (2.46-3.01)	5311.11 (4381.86-6343.84)	8.07 (6.66-9.58)	3.83 (3.71-3.96)	61.74	< 0.001
Western Sub-Saharan Africa	1552.51 (1402.68-1707.77)	1.47 (1.35-1.62)	3767.95 (3405.67-4156.05)	1.49 (1.36-1.62)	0.04 (0.02-0.05)	5.23	< 0.001

NRVHD= Non-rheumatic valvular heart disease; AAPC=average annual percentage change; UI=uncertain interval

Table 2 Number of NRVHD patients, age-standardized prevalence rates, and age-standardized average annual percent change, from 1999 to 2019

	1990		2019		1990-2019		
	Cases (95% *UI*)	ASR(per 100 000) (95% *UI*)	Cases (95% *UI*)	ASR(per 100 000) (95% *UI*)	AAPC (95% *CI*)	*t*	*P*
SDI							
High SDI	9394635 (8977145-9831626)	931.45 (890.04-975.62)	17791750 (16920830-18724020)	1035.03 (986.91-1086.88)	0.40 (0.30-0.40)	7.82	< 0.001
High-middle SDI	5034244 (4762268-5327488)	466.09 (440.95-493.25)	11361390 (10681960-12047770)	558.00 (524.91-591.39)	0.61 (0.55-0.67)	19.27	< 0.001
Middle SDI	865668.60 (793617.80-940547.40)	81.09 (74.45-87.79)	2824731 (2605221-3035513)	111.21 (102.84-119.24)	1.1 (1.07-1.13)	66.48	< 0.001
Low-middle SDI	196806.90 (174487.10-222250.70)	31.59 (28.01-35.56)	536706.70 (484104.90-591033.00)	38.09 (34.46-41.86)	0.64 (0.58-0.69)	21.80	< 0.001
Low SDI	31099.56 (26358.69-36899.46)	11.65 (9.85-13.74)	76163.54 (64999.06-89852.37)	12.72 (10.80-14.88)	1.1 (1.06-1.15)	48.04	< 0.001
Location							
Central Asia	204399.75 (166259.09-249918.64)	418.78 (343.29-511.42)	437310.24 (365454.77-522405.16)	551.18 (464.08-655.15)	0.97 (0.88-1.07)	20.87	< 0.001
Central Europe	1054901.75 (944376.93-1196050.11)	716.30 (641.58-809.87)	2622762.70 (2378271.54-2885848.57)	1292.65 (1174.74-1423.21)	2.08 (1.95-2.22)	30.51	< 0.001
Eastern Europe	1358196.58 (1279406.61-1436672.10)	494.42 (465.75-522.80)	2814546.22 (2550695.80-3088361.06)	857.81 (780.55-938.26)	1.95 (1.87-2.04)	46.38	< 0.001
Australasia	64866.82	275.95	417531.13	857.72	4.00	110.61	< 0.001

	(58562.54-72395.28)	(248.80-308.06)	(369612.01-479382.91)	(761.58-982.82)	(3.93-4.07)		
High-income Asia Pacific	3048522.24 (2904658.34-3197171.25)	1497.03 (1426.97-1569.33)	5882509.61 (5568283.30-6237461.09)	1553.60 (1471.19-1643.50)	0.12 (0.09-0.16)	6.78	< 0.001
High-income North America	5224300.98 (4992722.73-5473860.35)	1532.39 (1464.34-1603.78)	8086421.97 (7774722.26-8424525.71)	1438.37 (1383.56-1496.18)	-0.16 (-0.33-0.02)	-1.73	0.084
Southern Latin America	45713.43 (37383.38-55381.78)	98.49 (80.66-119.18)	179779.73 (156957.80-205085.08)	218.47 (191.34-249.27)	2.78 (2.71-2.84)	83.98	< 0.001
Western Europe	2625754.13 (2499751.66-2761641.45)	471.81 (449.74-495.72)	5356943.35 (5033805.75-5699575.26)	640.28 (604.88-678.83)	1.04 (0.89-1.19)	13.67	< 0.001
Andean Latin America	2477.00 (1920.98-3131.70)	11.05 (8.65-13.88)	36976.70 (31732.54-42758.57)	65.28 (55.98-75.36)	6.31 (6.00-6.63)	40.23	< 0.001
Caribbean	7196.78 (6131.19-8417.76)	26.61 (22.72-31.14)	52221.33 (44396.38-61526.97)	100.60 (85.51-118.35)	4.80 (4.37-5.24)	22.19	< 0.001
Central Latin America	15607.39 (13509.63-17892.81)	17.26 (14.89-19.83)	97861.87 (83421.54-111623.28)	40.23 (34.21-45.95)	2.99 (2.49-3.49)	11.91	< 0.001
Tropical Latin America	26719.26 (23814.08-29311.52)	25.11 (22.33-27.59)	97646.32 (84901.01-111593.10)	39.01 (33.91-44.59)	1.51 (1.39-1.64)	23.96	< 0.001
North Africa and Middle East	105801.42 (88463.70-126149.57)	54.18 (45.34-64.73)	314763.96 (268588.12-368045.15)	62.78 (53.71-73.52)	0.54 (0.44-0.64)	10.60	< 0.001

South Asia	56081.99 (49215.33-63309.32)	8.99 (7.82-10.07)	178959.74 (159248.68-200400.65)	11.88 (10.50-13.31)	0.95 (0.86-1.04)	21.35	< 0.001
East Asia	1532092.88 (1440421.96-1630071.66)	171.19 (161.33-181.92)	5544391.31 (5225685.38-5843184.96)	264.47 (249.97-278.46)	1.50 (1.48-1.53)	114.93	< 0.001
Oceania	2000.76 (1591.17-2466.46)	61.42 (49.56-75.00)	5545.56 (4506.90-6651.84)	74.32 (61.58-88.15)	0.65 (0.59-0.71)	22.38	< 0.001
Southeast Asia	121287.64 (104646.62-139656.32)	46.37 (40.14-53.31)	351927.11 (302126.81-406429.31)	56.53 (48.79-64.91)	0.68 (0.62-0.73)	23.90	< 0.001
Central Sub-Saharan Africa	3297.53 (2441.03-4294.30)	12.74 (9.56-16.45)	7110.41 (5308.41-9255.13)	11.42 (8.58-14.86)	-0.35 (-0.44--0.25)	-6.94	< 0.001
Eastern Sub-Saharan Africa	8724.47 (7170.32-10561.15)	10.28 (8.48-12.38)	19398.48 (15935.45-23452.35)	10.07 (8.35-12.16)	-0.08 (-0.15--0.004)	-2.06	0.04
Southern Sub-Saharan Africa	6406.02 (5644.11-7156.07)	20.88 (18.30-23.33)	64785.87 (51499.62-80284.09)	106.09 (84.25-131.29)	5.70 (4.90-6.50)	14.41	< 0.001
Western Sub-Saharan Africa	10996.77 (9410.97-12672.61)	10.69 (9.23-12.35)	28982.61 (25027.35-33572.17)	11.87 (10.24-13.78)	0.35 (0.31-0.39)	17.79	< 0.001

NRVHD= Non-rheumatic valvular heart disease; AAPC=average annual percentage change; UI=uncertain interval

Table 3 Number of NRVHD patients, age-standardized mortality rates, and age-standardized average annual percent change, from 1999 to 2019

	1990		2019		1990-2019		
	Cases （95%*UI*）	ASR(per 100 000) （95% *UI*）	Cases （95% *UI*）	ASR(per 100 000) （95% *UI*）	AAPC （95% *CI*）	*t*	*P*
SDI							
High SDI	46740.45 (42167.84-48979.31)	4.49 (4.02-4.71)	94294.70 (77325.12-104386.70)	3.99 (3.31-4.38)	-0.44 (-0.57--0.32)	-6.82	< 0.001
High-middle SDI	14094.00 (13130.27-14828.47)	1.54 (1.41-1.62)	32892.60 (28720.23-35781.67)	1.72 (1.49-1.87)	0.38 (0.25-0.50)	6.08	< 0.001
Middle SDI	8170.38 (7017.10-9080.79)	0.92 (0.79-1.01)	16850.25 (15233.37-18626.89)	0.77 (0.69-0.85)	-0.61 (-0.68--0.54)	-17.33	< 0.001
Low-middle SDI	5847.58 (4197.81-7421.06)	1.17 (0.86-1.44)	13684.21 (11337.08-15946.43)	1.14 (0.95-1.32)	-0.06 (-0.26-0.13)	-0.64	0.522
Low SDI	3036.43 (2055.45-4058.45)	1.46 (1.03-1.84)	6311.90 (4890.59-7535.87)	1.37 (1.10-1.62)	-0.25 (-0.33--0.17)	-6.40	< 0.001
Location							
Central Asia	140.08 (122.94-162.07)	0.31 (0.27-0.36)	422.92 (376.72-469.41)	0.66 (0.59-0.74)	2.85 (2.32-3.58)	10.71	< 0.001

Central Europe	2143.59 (2022.83-2254.90)	1.55 (1.46-1.64)	6834.63 (5823.56-7680.71)	3.06 (2.61-3.45)	2.38 (2.19-2.56)	25.34	< 0.001
Eastern Europe	934.78 (875.66-1003.76)	0.36 (0.33-0.38)	2251.48 (1956.66-2553.09)	0.67 (0.58-0.77)	2.32 (1.77-2.88)	8.30	< 0.001
Australasia	1015.08 (917.25-1070.73)	4.59 (4.10-4.87)	2237.36 (1838.58-2501.57)	3.85 (3.20-4.29)	-0.60 (-0.82--0.38)	-5.44	< 0.001
High-income Asia Pacific	6023.12 (5307.18-6368.19)	3.59 (3.11-3.82)	16604.12 (11447.87-19355.04)	2.45 (1.76-2.82)	-1.32 (-1.46--1.18)	-18.78	< 0.001
High-income North America	16470.43 (14618.68-17391.75)	4.46 (3.97-4.71)	31374.53 (26065.31-34627.25)	4.25 (3.59-4.67)	-0.15 (-0.47-0.18)	-0.89	0.375
Southern Latin America	1555.32 (1392.59-1729.76)	3.82 (3.42-4.25)	3116.33 (2723.47-3425.65)	3.62 (3.17-3.98)	-0.14 (-0.43-0.15)	-0.96	0.338
Western Europe	28078.95 (25620.70-29412.11)	4.78 (4.33-5.02)	56574.56 (47286.91-62323.03)	4.82 (4.08-5.29)	0.02 (-0.11-0.16)	0.34	0.737
Andean Latin America	180.33 (145.03-213.84)	0.87 (0.70-1.02)	408.11 (332.98-488.28)	0.73 (0.60-0.88)	-0.54 (-0.84--0.24)	-3.51	0.001
Caribbean	385.55	1.50	751.12	1.46	-0.08 (-0.31-	-0.67	0.5

	(339.18-423.87)	(1.32-1.64)	(617.10-889.27)	(1.20-1.72)	0.15)		
Central Latin America	991.94 (944.49-1031.15)	1.21 (1.13-1.26)	2786.22 (2327.43-3290.07)	1.21 (1.01-1.42)	-0.02 (-0.39-0.34)	-0.13	0.898
Tropical Latin America	2224.03 (2125.42-2312.04)	2.52 (2.37-2.64)	4986.08 (4459.22-5378.59)	2.15 (1.91-2.32)	-0.46 (-0.56--0.37)	-9.67	< 0.001
North Africa and Middle East	3968.51 (3145.72-4614.28)	2.54 (2.04-2.97)	7163.59 (6286.22-8180.95)	1.81 (1.60-2.07)	-1.21 (-1.33--1.08)	-18.88	< 0.001
South Asia	5617.41 (3664.76-7476.74)	1.30 (0.88-1.66)	14333.55 (11360.23-17190.56)	1.21 (0.98-1.44)	-0.21 (-0.59-0.17)	-1.07	0.286
East Asia	4037.61 (3148.84-4868.75)	0.58 (0.46-0.69)	5990.98 (5126.23-7243.24)	0.34 (0.29-0.40)	-1.94 (-2.11--1.77)	-22.28	< 0.001
Oceania	29.19 (19.37-43.03)	1.19 (0.83-1.64)	64.12 (43.96-92.43)	1.07 (0.78-1.47)	-0.38 (-0.50--0.25)	-5.97	< 0.001
Southeast Asia	1166.73 (994.27-1435.95)	0.53 (0.45-0.63)	2632.95 (2248.11-3309.00)	0.50 (0.43-0.62)	-0.11 (-0.24-0.02)	-1.62	0.105
Central Sub-	346.03	1.85	743.48	1.70	-0.30 (-0.37--	-7.93	< 0.001

Saharan Africa	(251.90-463.86)	(1.43-2.33)	(563.95-981.36)	(1.34-2.18)	0.23)		
Eastern Sub-	1193.67	1.81	2140.12	1.53	-0.57 (-0.64--	-15.64	< 0.001
Saharan Africa	(903.08-1528.70)	(1.45-2.19)	(1800.38-2623.48)	(1.28-1.95)	0.50)		
Southern Sub-	391.43	1.46	645.67	1.35	-0.21 (-0.59-	-1.11	0.266
Saharan Africa	(349.55-438.50)	(1.29-1.66)	(576.67-719.78)	(1.19-1.50)	0.16)		
Western Sub-	1038.41	1.17	2062.65	1.02	-0.47 (-0.52--	-16.64	< 0.001
Saharan Africa	(633.76-1499.54)	(0.73-1.65)	(1521.47-2676.18)	(0.76-1.30)	0.41)		

NRVHD= Non-rheumatic valvular heart disease; AAPC=average annual percentage change; UI=uncertain interval

Table 4 Number of patients, age-standardized DALY rates, and age-standardized average annual percent change, from 1999 to 2019

	1990		2019		1990-2019		
	Cases (95%*UI*)	ASR(per 100 000) (95% *UI*)	Cases (95% *UI*)	ASR(per 100 000) (95% *UI*)	AAPC (95% *CI*)	*t*	*P*
SDI							
High SDI	806863.50 (748412.60-870523.70)	77.26 (71.76-83.25)	1217971.00 (1065445.00-1359138.00)	58.69 (52.16-65.88)	-0.93 (-1.05--0.81)	-15.38	< 0.001
High-middle SDI	357464.90 (326453.90-396218.40)	34.37 (31.35-38.16)	623374.70 (559953.90-710928.80)	31.84 (28.64-36.13)	-0.28 (-0.38--0.17)	-5.20	< 0.001
Middle SDI	244007.10 (207459.10-278067.00)	21.05 (18.04-23.60)	429443.60 (390122.00-478487.50)	17.51 (15.94-19.55)	-0.63 (-0.69--0.58)	-22.49	< 0.001
Low-middle SDI	165641.70 (115579.30-218790.50)	25.15 (17.98-32.11)	340888.70 (275551.20-409533.60)	24.03 (19.69-28.41)	-0.13 (-0.29-0.04)	-1.50	0.133
Low SDI	91773.99 (59256.55-130809.50)	33.28 (22.43-44.88)	180409.30 (133445.00-226577.20)	29.60 (22.69-35.68)	-0.41 (-0.46--0.36)	-15.70	< 0.001
Location							
Central Asia	5731.65 (4765.75-7194.23)	11.75 (9.62-15.03)	14791.98 (12509.76-17873.50)	20.16 (17.09-24.76)	1.95 (1.60-2.30)	11.07	< 0.001

Central Europe	57920.99 (52689.67-65982.54)	40.26 (36.62-45.75)	135967.53 (116986.49-157048.00)	64.04 (55.24-73.77)	1.63 (1.50-1.76)	24.45	< 0.001
Eastern Europe	34349.15 (28788.28-42634.74)	12.81 (10.77-15.81)	74012.57 (60645.59-92151.25)	22.99 (18.97-28.47)	2.10 (1.55-2.65)	7.57	< 0.001
Australasia	16929.11 (15839.96-17863.89)	73.24 (68.21-77.25)	29901.13 (25923.58-33588.48)	56.55 (49.56-63.54)	-0.92 (-1.15--0.69)	-7.78	< 0.001
High-income Asia Pacific	120923.44 (108287.47-138083.39)	64.45 (57.92-73.62)	213950.80 (171309.06-257425.85)	38.80 (31.51-47.02)	-1.76 (-1.92--1.60)	-21.45	< 0.001
High-income North America	306146.01 (280297.66-341907.19)	85.74 (78.74-95.48)	435693.68 (385549.17-498948.92)	66.23 (59.23-75.73)	-0.86 (-1.04--0.68)	-9.24	< 0.001
Southern Latin America	28571.82 (26009.86-31556.43)	64.17 (58.35-70.99)	48750.97 (44420.35-52769.23)	58.21 (53.13-62.90)	-0.24 (-0.4--0.08)	-2.97	0.003
Western Europe	461698.66 (430331.83-488097.68)	79.84 (74.53-84.16)	686311.63 (605270.40-751096.16)	66.84 (59.88-72.87)	-0.64 (-0.75--0.53)	-11.54	< 0.001
Andean Latin America	5385.38 (4275.16-6536.25)	21.36 (17.03-25.59)	10535.51 (8594.04-12804.33)	17.99 (14.68-21.76)	-0.62 (-0.90--0.33)	-4.28	< 0.001
Caribbean	10692.81	37.45	18230.07	35.68	-0.14 (-0.27-	-1.91	0.056

	(9224.38-12052.63)	(32.53-41.90)	(14825.27-21996.84)	(28.96-43.14)	0.004)		
Central Latin America	28064.46 (27043.14-29032.72)	28.65 (27.50-29.72)	65378.78 (54584.08-77913.45)	27.16 (22.67-32.32)	-0.19 (-0.69-0.3)	-0.76	0.449
Tropical Latin America	67270.25 (64914.80-69635.96)	62.16 (59.65-64.41)	106991.73 (99343.55-113905.60)	44.28 (40.98-47.21)	-1.13 (-1.34--0.93)	-10.88	< 0.001
North Africa and Middle East	117555.51 (91584.73-141880.54)	57.90 (45.61-68.07)	194179.95 (167307.81-226089.19)	40.43 (35.07-46.60)	-1.24 (-1.32--1.16)	-30.76	< 0.001
South Asia	153771.72 (96669.41-212836.40)	25.81 (16.81-34.27)	337990.00 (258622.84-418855.35)	23.72 (18.56-28.84)	-0.27 (-0.61-0.07)	-1.55	0.12
East Asia	124098.42 (96942.20-152036.51)	13.85 (10.98-16.65)	183634.10 (152479.66-226235.71)	9.50 (7.96-11.71)	-1.32 (-1.41--1.23)	-27.84	< 0.001
Oceania	1015.59 (659.97-1559.15)	26.77 (18.21-38.66)	2212.98 (1456.23-3315.64)	25.05 (17.34-35.89)	-0.26 (-0.43--0.09)	-3.02	0.002
Southeast Asia	32862.85 (27701.25-41402.19)	11.72 (10.04-14.44)	65960.66 (56422.05-83995.02)	10.86 (9.30-13.61)	-0.24 (-0.38--0.10)	-3.29	0.001
Central Sub-	10190.59	39.87	20649.13	34.69	-0.48 (-0.58--	-9.71	< 0.001

Saharan Africa	(6945.02-14668.80)	(29.04-53.26)	(14734.99-27964.21)	(26.23-45.86)	0.39)		
Eastern Sub-Saharan Africa	36431.26 (25947.15-50421.47)	39.91 (29.97-51.64)	60137.19 (47914.64-73660.09)	30.81 (25.84-37.68)	-0.89 (-0.94--0.84)	-35.75	< 0.001
Southern Sub-Saharan Africa	12376.78 (10872.24-13748.27)	34.71 (30.94-38.80)	16822.18 (15029.28-19259.84)	27.65 (24.84-31.18)	-0.72 (-0.96--0.47)	-5.67	< 0.001
Western Sub-Saharan Africa	34719.33 (20660.35-51103.53)	31.62 (19.33-45.47)	71647.41 (51601.51-95353.97)	27.13 (20.07-35.23)	-0.51 (-0.59--0.43)	-12.64	< 0.001

NRVHD= Non-rheumatic valvular heart disease; AAPC=average annual percentage change; UI=uncertain interval

Table 5 Deaths and DALYs age-standardized rates of NRVHD attributable to all risk factors(diet high in sodium, lead exposure, high systolic blood pressure) in Global from 1990 to 2019.

Year	ASR(per 100,000) (95% *UI*)							
	All risk factors		Diet high in sodium		Lead exposure		High systolic blood pressure	
	Death	DALYs	Death	DALYs	Death	DALYs	Death	DALYs
1990	0.69 (0.49-0.95)	11.17 (8.37-14.36)	0.06 (0.01-0.17)	1.15 (0.26-2.89)	0.03 (0.01-0.05)	0.53 (0.23-0.88)	0.64 (0.46-0.85)	10.20 (7.82-12.86)
1991	0.70 (0.50-0.97)	11.23 (8.38-14.44)	0.06 (0.01-0.17)	1.16 (0.27-2.92)	0.03 (0.01-0.05)	0.53 (0.23-0.89)	0.65 (0.47-0.87)	10.25 (7.86-12.93)
1992	0.71 (0.50-0.99)	11.29 (8.46-14.53)	0.06 (0.01-0.17)	1.16 (0.27-2.91)	0.03 (0.01-0.05)	0.53 (0.23-0.89)	0.66 (0.47-0.88)	10.32 (7.90-13.02)
1993	0.72 (0.51-1.01)	11.41 (8.52-14.78)	0.06 (0.01-0.17)	1.16 (0.26-2.96)	0.03 (0.01-0.05)	0.54 (0.24-0.90)	0.67 (0.48-0.90)	10.43 (8.00-13.20)
1994	0.73 (0.51-1.01)	11.36 (8.53-14.75)	0.06 (0.01-0.17)	1.15 (0.26-2.93)	0.03 (0.01-0.05)	0.54 (0.24-0.91)	0.67 (0.48-0.90)	10.38 (7.97-13.13)
1995	0.73 (0.51-1.02)	11.28 (8.46-14.70)	0.06 (0.01-0.17)	1.13 (0.25-2.89)	0.03 (0.01-0.05)	0.54 (0.24-0.90)	0.67 (0.4800.91)	10.31 (7.93-13.10)

1996	0.72 (0.51-1.01)	11.13 (8.37-14.54)	0.06 (0.01-0.17)	1.11 (0.24-2.84)	0.03 (0.01-0.05)	0.54 (0.24-0.90)	0.67 (0.48-0.91)	10.17 (7.79-12.91)
1997	0.72 (0.50-1.01)	10.96 (8.24-14.29)	0.06 (0.01-0.17)	1.09 (0.24-2.82)	0.03 (0.01-0.05)	0.54 (0.24-0.89)	0.66 (0.47-0.90)	10.01 (7.65-12.70)
1998	0.71 (0.50-1.01)	10.82 (8.10-14.11)	0.06 (0.01-0.16)	1.08 (0.23-2.79)	0.03 (0.01-0.05)	0.53 (0.24-0.88)	0.66 (0.47-0.90)	9.88 (7.56-12.55)
1999	0.70 (0.49-0.99)	10.58 (7.91-13.79)	0.06 (0.01-0.16)	1.06 (0.23-2.76)	0.03 (0.01-0.05)	0.52 (0.24-0.86)	0.64 (0.46-0.88)	9.65 (7.37-12.30)
2000	0.69 (0.48-0.98)	10.36 (7.75-13.53)	0.06 (0.01-0.16)	1.04 (0.22-2.71)	0.03 (0.01-0.05)	0.51 (0.24-0.84)	0.64 (0.45-0.88)	9.45 (7.22-12.05)
2001	0.68 (0.47-0.96)	10.17 (7.59-13.33)	0.06 (0.01-0.16)	1.03 (0.22-2.65)	0.03 (0.01-0.05)	0.51 (0.24-0.84)	0.63 (0.44-0.87)	9.27 (7.08-11.82)
2002	0.68 (0.47-0.96)	10.05 (7.49-13.25)	0.06 (0.01-0.16)	1.02 (0.22-2.64)	0.03 (0.01-0.05)	0.50 (0.23-0.83)	0.62 (0.44-0.86)	9.15 (6.95-11.72)
2003	0.67 (0.46-0.96)	9.90 (7.36-13.10)	0.06 (0.01-0.16)	1.01 (0.22-2.61)	0.03 (0.01-0.05)	0.50 (0.23-0.82)	0.61 (0.43-0.85)	9.01 (6.84-11.60)
2004	0.65	9.67	0.06	1.00	0.03	0.48	0.60	8.79

	(0.44-0.94)	(7.16-12.82)	(0.01-0.15)	(0.21-2.57)	(0.01-0.05)	(0.23-0.80)	(0.42-0.83)	(6.65-11.31)
2005	0.65<br>(0.44-0.94)	9.60<br>(7.08-12.78)	0.06<br>(0.01-0.15)	1.00<br>(0.21-2.56)	0.03<br>(0.01-0.05)	0.48<br>(0.23-0.80)	0.60<br>(0.42-0.83)	8.72<br>(6.60-11.24)
2006	0.65<br>(0.43-0.94)	9.49<br>(6.99-12.67)	0.06<br>(0.01-0.15)	0.99<br>(0.21-2.54)	0.03<br>(0.01-0.05)	0.48<br>(0.22-0.79)	0.59<br>(0.41-0.83)	8.61<br>(6.55-11.09)
2007	0.65<br>(0.43-0.94)	9.44<br>(6.95-12.61)	0.06<br>(0.01-0.15)	0.99<br>(0.21-2.52)	0.03<br>(0.01-0.05)	0.47<br>(0.22-0.79)	0.59<br>(0.41-0.83)	8.56<br>(6.48-10.98)
2008	0.65<br>(0.43-0.94)	9.40<br>(6.93-12.57)	0.06<br>(0.01-0.16)	0.99<br>(0.20-2.53)	0.03<br>(0.01-0.05)	0.47<br>(0.22-0.78)	0.59<br>(0.41-0.83)	8.53<br>(6.47-10.95)
2009	0.64<br>(0.43-0.93)	9.32<br>(6.86-12.48)	0.06<br>(0.01-0.16)	0.99<br>(0.20-2.52)	0.03<br>(0.01-0.05)	0.46<br>(0.22-0.77)	0.59<br>(0.40-0.83)	8.45<br>(6.41-10.87)
2010	0.64<br>(0.42-0.93)	9.29<br>(6.78-12.40)	0.06<br>(0.01-0.16)	0.99<br>(0.21-2.52)	0.03<br>(0.01-0.05)	0.46<br>(0.21-0.77)	0.59<br>(0.40-0.83)	8.42<br>(6.35-10.81)
2011	0.64<br>(0.42-0.94)	9.29<br>(6.79-12.37)	0.06<br>(0.01-0.16)	1.00<br>(0.20-2.53)	0.03<br>(0.01-0.05)	0.46<br>(0.21-0.76)	0.59<br>(0.40-0.84)	8.42<br>(6.32-10.80)
2012	0.64<br>(0.43-0.94)	9.28<br>(6.77-12.40)	0.06<br>(0.01-0.16)	1.00<br>(0.21-2.52)	0.03<br>(0.01-0.05)	0.45<br>(0.21-0.75)	0.59<br>(0.40-0.84)	8.41<br>(6.29-10.79)

2013	0.64<br>(0.43-0.94)	9.25<br>(6.76-12.33)	0.06<br>(0.01-0.16)	1.00<br>(0.20-2.54)	0.03<br>(0.01-0.05)	0.45<br>(0.21-0.75)	0.59<br>(0.40-0.84)	8.38<br>(6.25-10.74)
2014	0.64<br>(0.42-0.93)	9.19<br>(6.70-12.16)	0.06<br>(0.01-0.16)	1.00<br>(0.20-2.52)	0.03<br>(0.01-0.05)	0.44<br>(0.20-0.74)	0.59<br>(0.39-0.83)	8.32<br>(6.20-10.73)
2015	0.64<br>(0.42-0.92)	9.21<br>(6.70-12.22)	0.06<br>(0.01-0.16)	1.00<br>(0.20-2.52)	0.03<br>(0.01-0.05)	0.44<br>(0.20-0.73)	0.58<br>(0.39-0.83)	8.34<br>(6.24-10.70)
2016	0.63<br>(0.41-0.91)	9.13<br>(6.70-12.09)	0.06<br>(0.01-0.16)	1.00<br>(0.20-2.51)	0.03<br>(0.01-0.05)	0.43<br>(0.20-0.72)	0.57<br>(0.38-0.82)	8.27<br>(6.20-10.60)
2017	0.61<br>(0.40-0.89)	8.99<br>(6.57-11.78)	0.06<br>(0.01-0.16)	0.99<br>(0.20-2.47)	0.03<br>(0.01-0.05)	0.42<br>(0.19-0.71)	0.56<br>(0.38-0.79)	8.14<br>(6.14-10.37)
2018	0.60<br>(0.40-0.87)	8.92<br>(6.56-11.75)	0.06<br>(0.01-0.16)	0.98<br>(0.20-2.48)	0.03<br>(0.01-0.05)	0.41<br>(0.19-0.70)	0.55<br>(0.37-0.78)	8.09<br>(6.06-10.31)
2019	0.60<br>(0.39-0.85)	8.86<br>(6.50-11.59)	0.06<br>(0.01-0.15)	0.98<br>(0.20-2.44)	0.03<br>(0.01-0.05)	0.40<br>(0.18-0.68)	0.54<br>(0.37-0.76)	8.03<br>(6.07-10.21)

Table 6 Deaths rates of NRVHD attributable to all risk factors(diet high in sodium, lead exposure, high systolic blood pressure) by age in Global from 1990 to 2019.

	Rates(per 100,000) (95% *UI*)							
	All risk factors		Diet high in sodium		Lead exposure		High systolic blood pressure	
	1990	2019	1990	2019	1990	2019	1990	2019
<20 years	0	0	0	0	0	0	0	0
20-54 years	0.08 (0.05-0.11)	0.07 (0.05-0.10)	0.01 (0.00-0.03)	0.01 (0.00-0.02)	0.01 (0.00-0.01)	0.00 (0.00-0.01)	0.07 (0.05-0.09)	0.07 (0.05-0.09)
55+ years	1.58 (1.15-2.12)	1.49 (0.98-2.14)	0.14 (0.03-0.38)	0.14 (0.02-0.38)	0.06 (0.03-0.11)	0.07 (0.03-0.12)	1.46 (1.07-1.91)	1.36 (0.92-1.91)
**Male**								
<20 years	0	0	0	0	0	0	0	0
20-54 years	0.12 (0.08-0.16)	0.11 (0.08-0.15)	0.02 (0.00-0.04)	0.02 (0.00-0.04)	0.01 (0.00-0.02)	0.01 (0.00-0.01)	0.10 (0.07-0.13)	0.10 (0.07-0.13)
55+ years	1.61 (1.21-2.10)	1.40 (0.97-1.95)	0.18 (0.04-0.44)	0.17 (0.03-0.42)	0.09 (0.04-0.14)	0.08 (0.04-0.13)	1.45 (1.10-1.86)	1.25 (0.87-1.67)
**Female**								

<20 years	0	0	0	0	0	0	0	0
20-54 years	0.04 (0.02-0.07)	0.04 (0.03-0.06)	0.01 (0.00-0.01)	0.00 (0.00-0.01)	0.00 (0.00-0.01)	0.00 (0.00-0.00)	0.04 (0.02-0.06)	0.04 (0.02-0.05)
55+ years	1.56 (1.10-2.20)	1.56 (0.98-2.34)	0.11 (0.02-0.33)	0.12 (0.02-0.36)	0.05 (0.02-0.09)	0.05 (0.02-0.11)	1.47 (1.03-2.00)	1.45 (0.93-2.14)

Table 7 DALY rates of NRVHD attributable to all risk factors(diet high in sodium, lead exposure, high systolic blood pressure) by age in Global from 1990 to 2019.

	Rates(per 100,000) (95% *UI*)							
	All risk factors		Diet high in sodium		Lead exposure		High systolic blood pressure	
	1990	2019	1990	2019	1990	2019	1990	2019
<20 years	0	0	0	0	0	0	0	0
20-54 years	3.61 (2.31-5.06)	3.37 (2.32-4.62)	0.51 (0.13-1.19)	0.45 (0.11-1.05)	0.26 (0.09-0.48)	0.15 (0.05-0.29)	3.16 (2.13-4.27)	3.04 (2.16-4.03)
55+ years	25.92 (19.47-33.04)	20.71 (14.89-27.67)	2.60 (0.59-6.65)	2.23 (0.44-5.69)	1.14 (0.51-1.88)	0.96 (0.45-1.61)	23.80 (18.03-29.59)	18.76 (13.62-24.33)
**Male**								
<20 years	0	0	0	0	0	0	0	0
20-54 years	5.34 (3.52-7.22)	4.92 (3.41-6.63)	0.79 (0.21-1.79)	0.70 (0.17-1.58)	0.40 (0.15-0.71)	0.23 (0.08-0.43)	4.66 (3.19-6.23)	4.43 (3.19-5.87)
55+ years	29.95 (22.66-37.81)	22.72 (16.55-29.67)	3.61 (0.90-8.74)	3.01 (0.64-7.14)	1.67 (0.85-2.65)	1.28 (0.66-2.05)	26.96 (20.69-33.15)	20.14 (14.97-25.32)
**Female**								

<20 years	0	0	0	0	0	0	0	0
20-54 years	1.83 (1.02-3.29)	1.79 (1.13-2.69)	0.23 (0.04-0.66)	0.20 (0.04-0.53)	0.11 (0.03-0.29)	0.07 (0.02-0.16)	1.62 (0.97-2.76)	1.63 (1.06-2.37)
55+ years	22.75 (16.69-30.36)	19.01 (12.92-26.65)	1.81 (0.33-5.06)	1.57 (0.24-4.58)	0.72 (0.23-1.39)	0.69 (0.26-1.30)	21.31 (15.57-28.34)	17.61 (12.23-23.82)

Table 8 Deaths age-standardized rates of NRVHD attributable to all risk factors(diet high in sodium, lead exposure, high systolic blood pressure) by sex and location in Global from 1990 to 2019.

	ASR(per 100,000) (95% *UI*)							
	All risk factors		Diet high in sodium		Lead exposure		High systolic blood pressure	
	1990	2019	1990	2019	1990	2019	1990	2019
Sex								
Male	0.74	0.63	0.08	0.07	0.04	0.04	0.67	0.57
	(0.54-1.00)	(0.43-0.89)	(0.02-0.20)	(0.01-0.19)	(0.02-0.06)	(0.02-0.06)	(0.50-0.88)	(0.39-0.78)
Female	0.63	0.55	0.05	0.04	0.02	0.02	0.59	0.51
	(0.43-0.90)	(0.35-0.81)	(0.01-0.14)	(0.01-0.13)	(0.01-0.04)	(0.01-0.04)	(0.41-0.82)	(0.33-0.75)
SDI								
High SDI	1.41	1.10	0.11	0.09	0.04	0.03	1.32	1.01
	(0.99-1.90)	(0.70-1.62)	(0.02-0.30)	(0.01-0.28)	(0.01-0.08)	(0.01-0.07)	(0.95-1.76)	(0.67-1.46)
High-middle	0.38	0.45	0.04	0.05	0.01	0.02	0.34	0.41
SDI	(0.27-0.50)	(0.31-0.62)	(0.01-0.10)	(0.01-0.12)	(0.01-0.03)	(0.01-0.03)	(0.25-0.45)	(0.29-0.55)
Middle SDI	0.18	0.19	0.03	0.02	0.01	0.01	0.15	0.17

	(0.24-0.13)	(0.25-0.14)	(0.01-0.06)	(0.01-0.05)	(0.01-0.02)	(0.01-0.02)	(0.11-0.20)	(0.13-0.21)
Low-middle	0.24	0.27	0.03	0.03	0.03	0.03	0.21	0.24
SDI	(0.35-0.15)	(0.38-0.19)	(0.00-0.07)	(0.00-0.07)	(0.01-0.04)	(0.02-0.05)	(0.13-0.30)	(0.17-0.32)
Low SDI	0.28	0.31	0.03	0.03	0.03	0.03	0.24	0.27
	(0.15-0.43)	(0.21-0.44)	(0.00-0.10)	(0.00-0.09)	(0.02-0.05)	(0.02-0.05)	(0.14-0.36)	(0.19-0.37)
Location								
Central Asia	0.06	0.14	0.01	0.01	0.00	0.00	0.06	0.13
	(0.05-0.08)	(0.10-0.20)	(0.00-0.02)	(0.00-0.04)	(0.00-0.00)	(0.00-0.01)	(0.04-0.07)	(0.09-0.18)
Central Europe	0.36	0.88	0.10	0.20	0.01	0.02	0.31	0.75
	(0.27-0.47)	(0.60-1.21)	(0.05-0.17)	(0.06-0.40)	(0.00-0.02)	(0.01-0.05)	(0.23-0.40)	(0.51-1.01)
Eastern Europe	0.09	0.21	0.01	0.02	0.00	0.00	0.09	0.20
	(0.07-0.12)	(0.15-0.28)	(0.00-0.02)	(0.00-0.05)	(0.00-0.00)	(0.00-0.01)	(0.06-0.11)	(0.14-0.25)
Australasia	1.37	0.98	0.05	0.04	0.06	0.05	1.30	0.92
	(0.98-1.85)	(0.62-1.44)	(0.01-0.20)	(0.01-0.16)	(0.03-0.11)	(0.02-0.10)	(0.93-1.75)	(0.59-1.34)
High-income	1.13	0.62	0.22	0.09	0.02	0.01	1.00	0.55
Asia Pacific	(0.76-1.58)	(0.35-0.98)	(0.06-0.45)	(0.01-0.25)	(0.00-0.04)	(0.00-0.03)	(0.68-1.39)	(0.32-0.86)
High-income	1.37	1.08	0.08	0.09	0.06	0.04	1.30	1.00

North America	(0.96-1.88)	(0.67-1.61)	(0.01-0.26)	(0.01-0.28)	(0.02-0.10)	(0.01-0.08)	(0.92-1.71)	(0.64-1.43)
Southern Latin	0.99	1.12	0.13	0.12	0.02	0.03	0.88	1.02
America	(0.64-1.45)	(0.76-1.59)	(0.01-0.37)	(0.01-0.35)	(0.00-0.06)	(0.00-0.06)	(0.61-1.24)	(0.70-1.41)
Western	1.49	1.40	0.09	0.09	0.04	0.04	1.42	1.32
Europe	(1.09-2.00)	(0.93-2.04)	(0.01-0.28)	(0.01-0.29)	(0.01-0.08)	(0.01-0.09)	(1.05-1.87)	(0.88-1.88)
Andean Latin	0.15	0.16	0.02	0.02	0.01	0.01	0.12	0.14
America	(0.09-0.22)	(0.11-0.24)	(0.00-0.07)	(0.00-0.06)	(0.01-0.02)	(0.00-0.02)	(0.08-0.17)	(0.10-0.19)
Caribbean	0.36	0.37	0.03	0.03	0.03	0.03	0.32	0.33
	(0.26-0.49)	(0.25-0.50)	(0.00-0.09)	(0.00-0.09)	(0.02-0.05)	(0.02-0.05)	(0.24-0.42)	(0.24-0.44)
Central Latin	0.31	0.34	0.05	0.05	0.03	0.03	0.27	0.30
America	(0.23-0.41)	(0.24-0.48)	(0.01-0.11)	(0.01-0.12)	(0.02-0.04)	(0.01-0.04)	(0.20-0.33)	(0.21-0.40)
Tropical Latin	0.72	0.61	0.08	0.06	0.04	0.03	0.65	0.55
America	(0.52-0.94)	(0.43-0.81)	(0.01-0.22)	(0.01-0.18)	(0.02-0.06)	(0.01-0.05)	(0.48-0.80)	(0.40-0.71)
North Africa	0.41	0.37	0.01	0.01	0.04	0.03	0.38	0.34
and Middle	(0.27-0.59)	(0.27-0.49)	(0.00-0.05)	(0.00-0.04)	(0.02-0.06)	(0.02-0.05)	(0.25-0.55)	(0.26-0.45)
East								

South Asia	0.27	0.29	0.02	0.02	0.04	0.04	0.23	0.25
	(0.15-0.41)	(0.19-0.41)	(0.00-0.08)	(0.00-0.07)	(0.02-0.06)	(0.02-0.06)	(0.13-0.35)	(0.17-0.34)
East Asia	0.08	0.08	0.03	0.02	0.01	0.01	0.06	0.06
	(0.04-0.12)	(0.05-0.10)	(0.01-0.05)	(0.01-0.04)	(0.00-0.01)	(0.00-0.01)	(0.03-0.08)	(0.04-0.08)
Oceania	0.20	0.21	0.04	0.04	0.01	0.01	0.17	0.17
	(0.11-0.33)	(0.12-0.32)	(0.01-0.10)	(0.00-0.10)	(0.00-0.01)	(0.00-0.01)	(0.10-0.25)	(0.11-0.26)
Southeast Asia	0.13	0.14	0.03	0.02	0.00	0.00	0.11	0.12
	(0.09-0.18)	(0.10-0.19)	(0.01-0.06)	(0.00-0.05)	(0.00-0.01)	(0.00-0.01)	(0.08-0.15)	(0.09-0.17)
Central Sub-	0.39	0.40	0.02	0.02	0.02	0.02	0.37	0.37
Saharan Africa	(0.24-0.61)	(0.27-0.59)	(0.00-0.08)	(0.00-0.08)	(0.01-0.04)	(0.01-0.04)	(0.22-0.57)	(0.25-0.54)
Eastern Sub-	0.33	0.36	0.07	0.05	0.03	0.03	0.26	0.31
Saharan Africa	(0.19-0.52)	(0.24-0.53)	(0.01-0.19)	(0.00-0.15)	(0.01-0.05)	(0.01-0.04)	(0.17-0.40)	(0.22-0.45)
Southern Sub-	0.37	0.38	0.02	0.02	0.01	0.01	0.35	0.37
Saharan Africa	(0.27-0.50)	(0.28-0.53)	(0.00-0.09)	(0.00-0.08)	(0.01-0.03)	(0.01-0.03)	(0.26-0.47)	(0.26-0.49)
Western Sub-	0.22	0.24	0.02	0.02	0.01	0.01	0.20	0.23
Saharan Africa	(0.11-0.37)	(0.15-0.35)	(0.00-0.07)	(0.00-0.07)	(0.00-0.03)	(0.01-0.02)	(0.11-0.33)	(0.15-0.32)

Table 9 DALY age-standardized rates of NRVHD attributable to all risk factors(diet high in sodium, lead exposure, high systolic blood pressure) by sex and location in Global from 1990 to 2019.

	ASR(per 100 000) (95% *UI*)							
	All risk factors		Diet high in sodium		Lead exposure		High systolic blood pressure	
	1990	2019	1990	2019	1990	2019	1990	2019
Sex								
Male	13.28	10.51	1.60	1.37	0.77	0.57	11.91	9.35
	(10.20-16.95)	(7.82-13.86)	(0.40-3.82)	(0.31-3.21)	(0.38-1.25)	(0.28-0.92)	(9.42-14.80)	(7.14-11.66)
Female	9.07	7.24	0.75	0.62	0.31	0.27	8.46	6.69
	(6.52-12.40)	(5.10-9.89)	(0.14-2.08)	(0.10-1.77)	(0.10-0.61)	(0.10-0.51)	(6.21-11.44)	(4.75-8.86)
SDI								
High SDI	22.84	14.98	1.93	1.43	0.69	0.38	21.42	13.84
	(17.25-28.80)	(10.59-20.34)	(0.37-5.12)	(0.19-3.95)	(0.21-1.31)	(0.09-0.82)	(16.12-26.53)	(9.90-18.19)
High-middle	7.37	8.02	1.02	1.08	0.30	0.27	6.64	7.24
SDI	(5.66-9.35)	(5.97-10.30)	(0.31-2.22)	(0.31-2.34)	(0.11-0.52)	(0.10-0.50)	(5.25-8.13)	(5.47-9.02)
Middle SDI	4.15	4.23	0.71	0.60	0.36	0.27	3.49	3.73

	(2.88-5.59)	(3.21-5.40)	(0.21-1.52)	(0.18-1.28)	(0.20-0.56)	(0.14-0.43)	(2.56-4.50)	(2.94-4.59)
Low-middle	5.05	5.63	0.57	0.60	0.64	0.58	4.30	4.94
SDI	(3.03-7.34)	(3.97-7.72)	(0.08-1.59)	(0.08-1.55)	(0.32-1.01)	(0.34-0.87)	(2.72-6.11)	(3.60-6.62)
Low SDI	6.17	6.49	0.73	0.61	0.70	0.67	5.27	5.73
	(3.24-9.77)	(4.31-9.27)	(0.05-2.33)	(0.04-1.85)	(0.32-1.19)	(0.38-1.03)	(2.92-8.04)	(3.92-7.88)
Location								
Central Asia	1.63	3.30	0.24	0.32	0.05	0.10	1.48	3.07
	(1.22-2.04)	(2.39-4.46)	(0.05-0.53)	(0.03-0.87)	(0.01-0.10)	(0.03-0.20)	(1.13-1.79)	(2.26-4.05)
Central Europe	8.25	18.00	2.29	4.06	0.19	0.43	7.11	15.50
	(6.41-10.35)	(12.65-23.67)	(1.09-3.76)	(1.29-7.70)	(0.04-0.38)	(0.09-0.90)	(5.58-8.83)	(11.22-19.57)
Eastern Europe	2.41	6.55	0.24	0.69	0.03	0.07	2.27	6.16
	(1.68-3.10)	(4.75-8.73)	(0.03-0.64)	(0.08-1.73)	(0.00-0.08)	(0.00-0.22)	(1.63-2.84)	(4.53-7.87)
Australasia	22.09	15.10	1.01	0.77	1.07	0.73	20.95	14.14
	(16.68-28.09)	(10.58-20.69)	(0.12-3.66)	(0.10-2.70)	(0.47-1.77)	(0.30-1.32)	(15.70-25.91)	(10.03-18.75)
High-income	18.13	8.40	3.84	1.33	0.29	0.14	15.94	7.44
Asia Pacific	(13.29-23.22)	(5.45-12.09)	(1.17-7.30)	(0.19-3.15)	(0.02-0.69)	(0.01-0.35)	(11.78-20.12)	(5.07-10.37)
High-income	22.98	14.73	1.37	1.39	0.96	0.47	21.62	13.48

North America	(17.05-29.61)	(10.21-20.46)	(0.13-4.58)	(0.13-4.15)	(0.35-1.72)	(0.11-0.97)	(16.33-26.71)	(9.69-18.02)
Southern Latin America	16.72<br>(11.48-23.17)	18.41<br>(13.26-24.40)	2.25<br>(0.17-6.21)	2.04<br>(0.14-5.63)	0.45<br>(0.02-1.04)	0.43<br>(0.06-0.95)	14.89<br>(10.99-19.56)	16.88<br>(12.44-21.40)
Western Europe	24.84<br>(19.19-31.18)	19.26<br>(13.83-25.72)	1.56<br>(0.16-4.87)	1.35<br>(0.14-4.11)	0.71<br>(0.22-1.35)	0.53<br>(0.15-1.07)	23.66<br>(18.53-29.02)	18.13<br>(13.23-23.69)
Andean Latin America	3.40<br>(1.95-5.34)	3.90<br>(2.58-5.50)	0.57<br>(0.03-1.69)	0.52<br>(0.04-1.45)	0.31<br>(0.12-0.57)	0.24<br>(0.10-0.44)	2.74<br>(1.79-3.94)	3.39<br>(2.38-4.49)
Caribbean	8.31<br>(6.00-10.83)	8.46<br>(5.86-11.47)	0.62<br>(0.04-2.04)	0.60<br>(0.04-1.97)	0.76<br>(0.42-1.15)	0.62<br>(0.32-0.99)	7.47<br>(5.65-9.42)	7.74<br>(5.51-10.31)
Central Latin America	7.60<br>(5.65-9.78)	7.85<br>(5.55-10.80)	1.19<br>(0.21-2.75)	1.11<br>(0.18-2.69)	0.71<br>(0.42-1.03)	0.51<br>(0.26-0.82)	6.44<br>(4.99-7.87)	6.91<br>(5.05-9.09)
Tropical Latin America	17.06<br>(12.72-21.63)	12.11<br>(8.95-15.66)	1.96<br>(0.14-5.28)	1.30<br>(0.11-3.55)	0.92<br>(0.38-1.54)	0.48<br>(0.18-0.87)	15.43<br>(12.11-18.69)	11.09<br>(8.45-13.72)
North Africa and Middle East	9.32<br>(6.08-13.03)	8.37<br>(6.18-10.74)	0.32<br>(0.05-1.24)	0.29<br>(0.06-1.08)	0.92<br>(0.48-1.50)	0.63<br>(0.34-1.00)	8.58<br>(5.70-11.85)	7.84<br>(5.92-9.94)
South Asia	5.29	5.58	0.50	0.55	0.75	0.69	4.50	4.83

	(2.88-7.99)	(3.77-7.83)	(0.04-1.56)	(0.05-1.51)	(0.37-1.21)	(0.41-1.04)	(2.52-6.66)	(3.37-6.61)
East Asia	2.02	2.04	0.77	0.64	0.20	0.13	1.40	1.60
	(1.17-3.06)	(1.46-2.71)	(0.30-1.39)	(0.29-1.10)	(0.11-0.34)	(0.07-0.21)	(0.82-2.06)	(1.17-2.09)
Oceania	3.84	4.28	0.68	0.65	0.10	0.09	3.27	3.77
	(2.11-6.24)	(2.48-6.70)	(0.10-1.80)	(0.07-1.73)	(0.01-0.27)	(0.01-0.23)	(1.95-5.00)	(2.36-5.62)
Southeast Asia	2.82	2.89	0.65	0.49	0.12	0.10	2.40	2.56
	(2.02-3.87)	(2.06-3.96)	(0.19-1.26)	(0.08-1.10)	(0.04-0.22)	(0.03-0.19)	(1.75-3.27)	(1.89-3.41)
Central Sub-	8.29	7.90	0.37	0.38	0.45	0.46	7.84	7.43
Saharan Africa	(4.73-13.83)	(5.19-11.94)	(0.03-1.65)	(0.04-1.61)	(0.16-0.93)	(0.21-0.81)	(4.50-12.81)	(4.94-10.93)
Eastern Sub-	6.89	7.04	1.42	0.94	0.61	0.44	5.56	6.25
Saharan Africa	(3.83-11.21)	(4.92-9.77)	(0.11-3.99)	(0.06-2.64)	(0.28-1.05)	(0.22-0.74)	(3.44-8.36)	(4.63-8.54)
Southern Sub-	8.03	7.51	0.62	0.43	0.31	0.28	7.56	7.13
Saharan Africa	(5.96-10.40)	(5.69-9.72)	(0.04-2.23)	(0.04-1.68)	(0.11-0.57)	(0.10-0.50)	(5.83-9.58)	(5.55-9.04)
Western Sub-	5.71	6.10	0.49	0.46	0.38	0.30	5.18	5.71
Saharan Africa	(2.80-9.53)	(3.82-8.97)	(0.02-2.05)	(0.02-1.74)	(0.12-0.76)	(0.12-0.56)	(2.68-8.46)	(3.70-8.23)

Table 10 Incidence, Deaths and DALYs projections of NRVHD in Global from 2020 to 2035.

Year	Incidence		Death		DALYs	
	Number	ASIR (per 100 000)	number	ASMR (per 100 000)	Number	ASDR(per 100 000)
2020	1706099	19.91	171622	2.27	2887888	36.02
2021	1754621	20.03	176674	2.26	2958227	35.86
2022	1803135	20.15	180653	2.24	3022400	35.70
2023	1845711	20.20	185024	2.23	3089985	35.54
2024	1888903	20.24	190430	2.21	3165429	35.37
2025	1932311	20.29	196071	2.19	3243559	35.21
2026	1975608	20.34	201484	2.18	3320995	35.05
2027	2018520	20.38	206418	2.16	3395558	34.89
2028	2055238	20.37	211996	2.15	3474868	34.74
2029	2091911	20.36	218428	2.13	3560948	34.60
2030	2128402	20.34	225233	2.12	3650124	34.45
2031	2164628	20.33	232077	2.11	3739644	34.31
2032	2200532	20.32	238722	2.09	3827371	34.17
2033	2236457	20.30	245757	2.08	3917729	34.02

2034	2272739	20.29	253590	2.07	4014230	33.88
2035	2309174	20.28	261819	2.06	4113473	33.74

NRVHD= Non-rheumatic valvular heart disease; ASIR=age standardized incidence rate; ASMR=age-standardized mortality rate; ASDR=age-standardized DALY rate

Table 11 Incidence, Deaths and DALYs projections of NRVHD by sex in Global from 2020 to 2035.

Year	Male						Female					
	Incidence		Death		DALYs		Incidence		Death		DALYs	
	Number	ASIR (per 100 000)	number	ASMR(per 100 000)	Number	ASDR(per 100 000)	Number	ASIR(per 100 000)	number	ASMR(per 100 000)	Number	ASDR(per 100 000)
2020	785083	18.96	72477	2.30	1381091	37.99	921016	20.79	99145	2.21	1506798	33.82
2021	810242	19.18	74785	2.29	1415640	37.87	944379	20.82	101889	2.19	1542587	33.64
2022	835536	19.39	76740	2.28	1447901	37.75	967599	20.86	103913	2.17	1574499	33.45
2023	857539	19.51	78824	2.27	1481128	37.61	988172	20.83	106200	2.15	1608857	33.28
2024	879958	19.63	81326	2.25	1517639	37.47	1008945	20.81	109105	2.14	1647789	33.11
2025	902624	19.75	83930	2.24	1555260	37.33	1029687	20.78	112141	2.12	1688299	32.94
2026	925404	19.87	86451	2.23	1592517	37.19	1050204	20.75	115034	2.10	1728478	32.77
2027	948191	20.00	88780	2.22	1628419	37.05	1070329	20.73	117638	2.08	1767139	32.60
2028	967092	20.03	91269	2.20	1665209	36.90	1088146	20.67	120727	2.07	1809659	32.47
2029	986201	20.07	94111	2.19	1704776	36.75	1105710	20.61	124317	2.06	1856172	32.33
2030	1005373	20.10	97095	2.18	1745445	36.61	1123029	20.55	128138	2.05	1904679	32.19
2031	1024494	20.14	100074	2.17	1785955	36.46	1140134	20.49	132003	2.03	1953689	32.06

2032	1043470	20.18	102940	2.15	1825325	36.31	1157062	20.43	135783	2.02	2002046	31.92
2033	1062519	20.21	105949	2.14	1865497	36.16	1173938	20.37	139808	2.01	2052232	31.79
2034	1081853	20.25	109277	2.13	1908062	36.02	1190886	20.31	144314	2.00	2106168	31.65
2035	1101296	20.29	112742	2.11	1951523	35.87	1207878	20.25	149076	1.98	2161949	31.52

ASIR=age standardized incidence rate; ASMR=age-standardized mortality rate; ASDR=age-standardized DALY rate

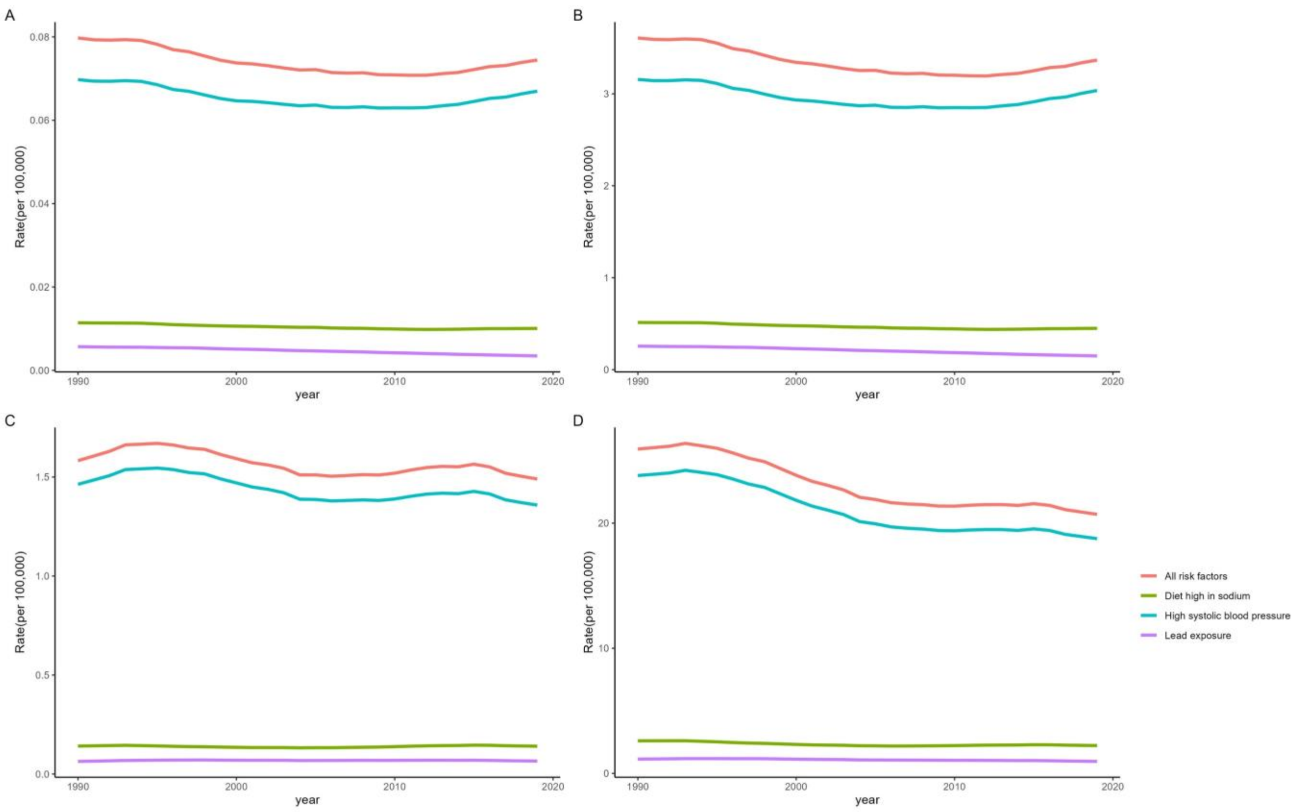

Supplementary Figure 1 Changes in Global NRVHD Risk Factors for Mortality (A), DALYs (B) in the 20-54 years group and Mortality (C), DALYs (D) in the 55+ years group from 1990 to 2019

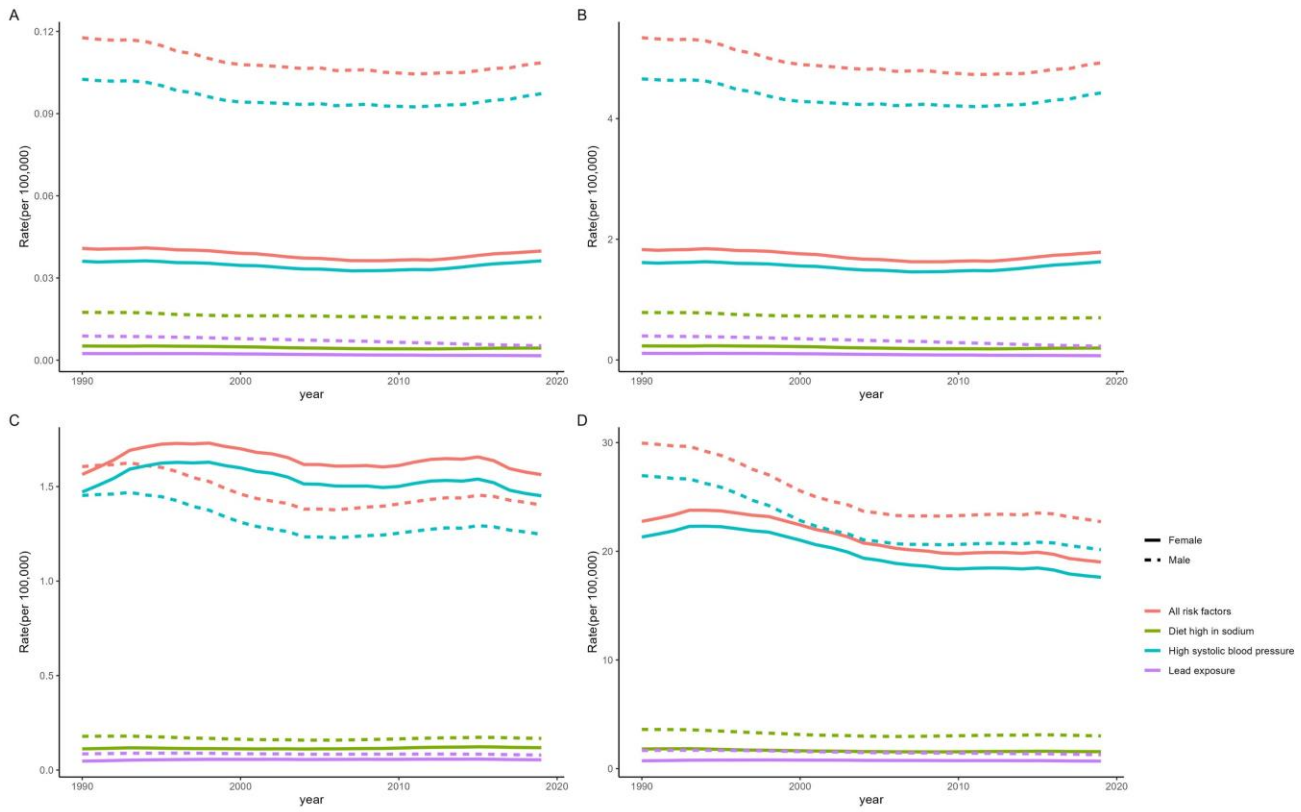

Supplementary Figure 2 Changes in Sex Differences in Mortality (A), DALYs (B) for 20-54 years group and Mortality (C), DALYs (D) for 55+ years group for global NRVHD risk factors from 1990 to 2019
